# Twisting of DNA Origami from Intercalators

**DOI:** 10.1038/s41598-017-07796-3

**Published:** 2017-08-07

**Authors:** Reza M. Zadegan, Elias G. Lindau, William P. Klein, Christopher Green, Elton Graugnard, Bernard Yurke, Wan Kuang, William L. Hughes

**Affiliations:** 1 Micron School of Materials Science & Engineering, Boise State University, Boise, Idaho 83725 United States; 2 Department of Electrical & Computer Engineering, Boise State University, Boise, Idaho 83725 United States

## Abstract

DNA nanostructures represent the confluence of materials science, computer science, biology, and engineering. As functional assemblies, they are capable of performing mechanical and chemical work. In this study, we demonstrate global twisting of DNA nanorails made from two DNA origami six-helix bundles. Twisting was controlled using ethidium bromide or SYBR Green I as model intercalators. Our findings demonstrate that DNA nanorails: (i) twist when subjected to intercalators and the amount of twisting is concentration dependent, and (ii) twisting saturates at elevated concentrations. This study provides insight into how complex DNA structures undergo conformational changes when exposed to intercalators and may be of relevance when exploring how intercalating drugs interact with condensed biological structures such as chromatin and chromosomes, as well as chromatin analogous gene expression devices.

## Introduction

Biologically, DNA encodes life *for* life by storing, organizing, and regulating genetic information to build and maintain vital ecosystems^1^. From materials science, nanotechnology, and engineering perspectives DNA is a functional material because of its ability to rationally self-assemble. Enabled by rapid progress in DNA synthesis, sophisticated DNA nanostructures^2–4^ represent the confluence of materials science, computer science, biology, and engineering. For example, DNA origami has been made into a diversity of 2D and 3D structures^4–6^, with applications that range from photonics^7^, chemical reactions^8^, and semiconductor fabrication^9^, to diagnostics^10, 11^, therapeutics^12^, and drug delivery^13, 14^. Among health-related applications, using DNA nanostructures for drug delivery of chemotherapeutic intercalators is of growing interest^12, 13, 15, 16^. Although few reports have been focused on studying these interactions^17–20^, more research is needed for understanding how intercalators induce conformational changes in DNA nanostructures such as chromatin analogous gene expression (CAGE) machines^21^.

Intercalators cause structural distortions in DNA by stacking in between base-pairs^22^. These observed distortions likely inhibit macromolecular biosynthesis and hence select intercalators are used for chemotherapy^23^. Chemotherapeutic intercalators inhibit DNA replication in rapidly growing cancer cells by inserting themselves between planar bases^23^. Insertion occurs because the drugs are hydrophobic and hence attempt to eliminate their interaction with water. Once inserted, unwinding of the DNA duplex occurs, the extent of which correlates to the concentration, shape, and size of the intercalator^22^. To eliminate off-target side effects, intercalators must site-specifically attack cancer cells. Towards this goal, a number of smart-delivery systems have been made using DNA origami as the vessel^12, 15, 21, 24–27^.

While intercalators are being explored in DNA nanotechnology and cancer research, few studies have been done to model their interaction with condensed DNA structures^17, 18, 21^. Presented here, we demonstrate concentration-dependent twisting of laddered, six-helix bundles (6HB), known as DNA nanorails^28^. Experimental results are in qualitative agreement with the McGhee-von Hippel theory as a model for ligand binding to a homogeneous linear polymer^29^. Our results provide a model system for controlling conformational changes in DNA nanostructures, as well as a model system for loading DNA nanomaterials with intercalators for downstream medical applications.

## Results

**Figure 1:**
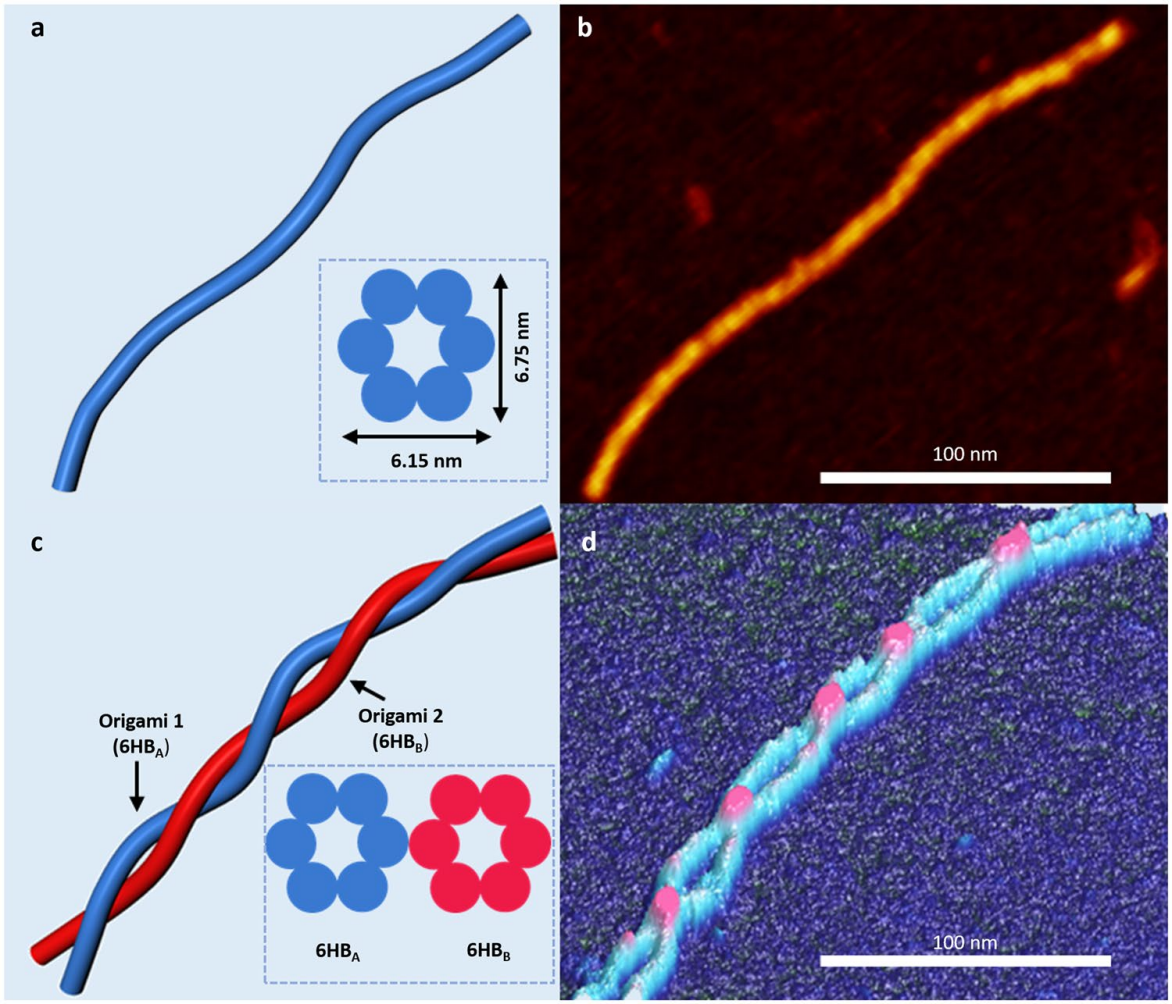
Schematic (**a**) and AFM image (**b**) of a DNA origami six-helix bundle (6HB). Inset in (**a**) illustrates the cross section of the bundle. Schematic (**c**) and AFM image (**d**) of a DNA nanorail twisting with left-handed chirality. Twisting was caused by ethidium bromide. Inset in (**c**) illustrates the nanorail cross section.

DNA origami six-helix bundles (6HB) were designed and synthesized using prior methods (Fig. 1a,b)^28^. The synthesized 420 nm nanorails included two independent 6HBs that were linked by nine double crossovers (Supporting Information S1). The crossovers were periodically spread across the length of the nanorail every 43 nm. The 6HBs (Fig. 1b) were independently synthesized and purified prior to cross-linking them together to form nanorails (Fig. 1c). Conformational changes in the DNA nanorails were measured via AFM as a function of the intercalator concentration from 0–30 µM. Ethidium bromide (Sigma Aldrich) and SYBR Green I (Sigma Aldrich) were adopted as model intercalators because they predictably lengthen duplex DNA when inserted between base-pairs^30, 31^. To accommodate for changes in length, DNA duplexes undergo left-handed helical unwinding^32, 33^. Unwinding caused left-handed global twisting of the nanorails (Fig. 1c), which was observed by measuring the number and chirality of cross-over points on atomically flat mica (Fig. 1d). Multiple AFM techniques were adopted to ensure that the chirality was not an imaging artifact (Supporting Information S2).

**Figure 2:**
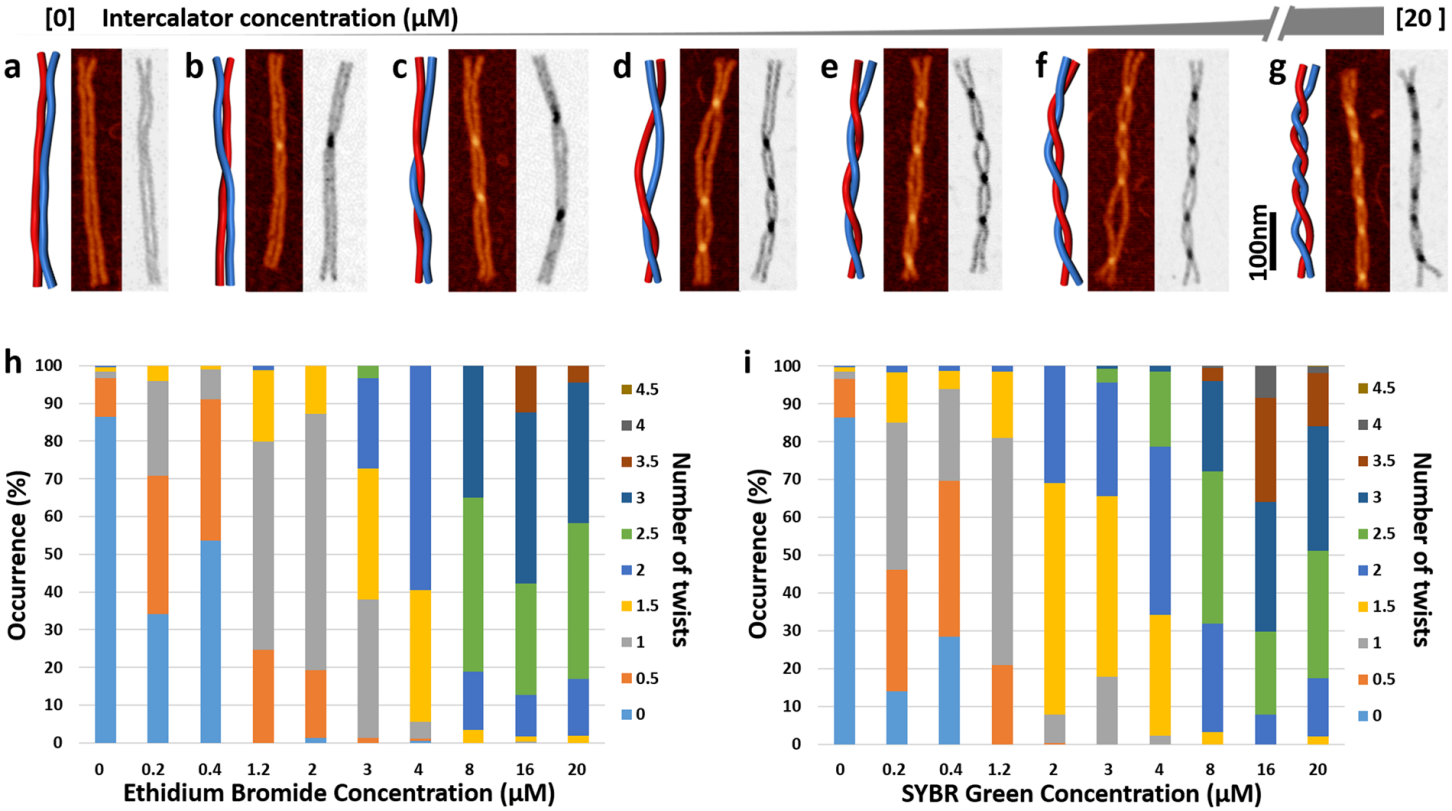
Illustrations and corresponding AFM images showing global twisting of DNA nanorails as a function of the dye concentration (EtBr is colored and SG is in black and white). Global twisting was not observed without EtBr or SG in (**a**) and increased as the [dye] increased from 200 nM (**b**) to 1.2 µM (**c**), 3 µM (**d**), 4 µM (**e**), 8 µM (**f**), and 16 µM (**g**). Summary of twisting effect of intercalators on DNA nanorails for samples treated with EtBr (**h**) and SG (**i**). Occurrence refers to the frequency of each individual state in the total number of particles counted for each data set.

**Figure 3:**
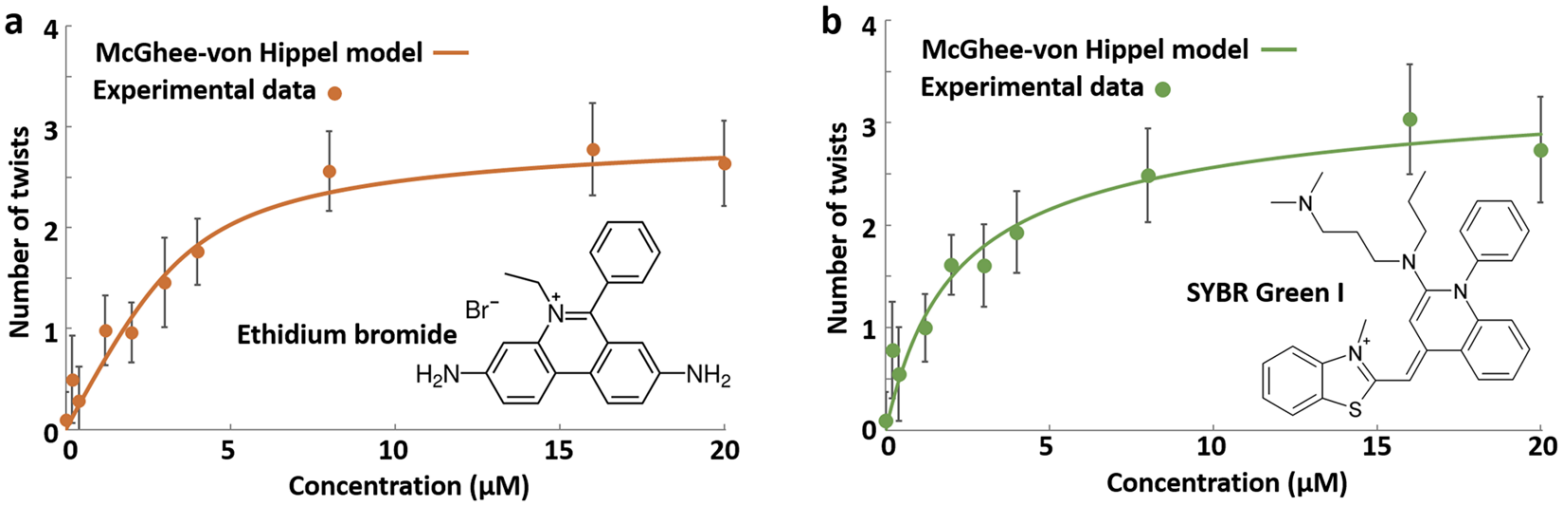
The number of twists in DNA origami nanorails as a function of the intercalator concentration for (**a**) ethidium bromide and (**b**) SYBR Green I. Filled circles are experimental data points and lines are fits using the McGhee-von Hippel model (Supporting Information S6). Error bars represent root-mean-square deviation of the data from the mean.

Left-handed global twisting of DNA nanorails increased as a function of the intercalator concentration (Fig. 2, Supporting Information S3 and S4). Both SYBR Green I (SG) and ethidium bromide (EtBr) promote nanostructure twisting and the number of twists increased as the concentration of the dyes increased. We denote the number of nanorail twists by *N*_*T*_, where one twist (*N*_*T*_ = 1) is a full rotation of its subunits (6HB_A_ and 6HB_B_). Thus, a single overlap on a nanorail AFM image (*brighter pixels for EtBr, darker pixels for SG*) is a half twist (*N*_*T*_ = ½). For a given intercalator concentration, at least 100 nanorails were analyzed. The average number of twists increase as a function of the intercalator concentration and seem to saturate at greater concentration of intercalator dyes (Fig. 3). AFM imaging of the twisted samples when treated at higher concentration of the intercalators than 30 μM, usually resulted in visualization of bare mica. To investigate whether the high pressure of the intercalation process caused sheering or denaturing of the DNA nanostructures, we performed qPCR studies on the M13mp18 scaffold and folded structures (Supporting Information S5). However, we did not observe any significant difference between intercalator treated and untreated samples to support the aforementioned possibilities.

Beyond 5 µM of dyes, turnover of the amount of twisting is likely caused by a decrease in the binding affinity between the intercalator and the DNA nanorail. Our assumption is consistent with previous reports indicating that intercalator binding blocks future binding of intercalating agents at neighboring sites^22, 34^. A full theory for dye intercalation in structures as complex as the DNA nanorail is not available for comparison with our experiment. However, the McGhee-von Hippel theory, which describes ligand binding on a homogeneous linear polymer^29^ provides a good theoretical model for comparison when each DNA duplex, that spans the length of the nanorail, is assumed as a continuous homogeneous polymer – with stacking interactions that maintain the B-form structure across the nick sites^29^. Nick sites are results of DNA nanorails being made by folding the scaffold strand into a geometrical arrangement that is bound together via stable strands – with multiple DNA duplexes that have a large number of crossovers and nicks. Nonetheless, the non-nicked segments of the nanorail are 16 nt in length, which is sufficient to accommodate several intercalating molecules.

Connection between the number of observed nanorail twists, *N*_*T*_ (or 2*π* radian turns), and the McGhee-von Hippel theory can be made by assuming a linear relation between the number of intercalators, *B*, and *N*_*T*_, that is, *N*_*T*_ = *αB*, where *α* is a proportionality constant. Two physical constants enter in the McGhee-von Hippel theory when cooperative effects are neglected in ligand binding. These are the rate constant, *K*, for the binding of a single ligand to a ligand binding site and the number of binding sites that are blocked, *n*, when a ligand binds. Given the concentration of the polymer, the McGhee-von Hipple theory gives *B* as a function of the total dye concentration. Although the results of McGhee-von Hipple theory are expressed in algebraic form, the expressions are complicated and generally must be dealt with numerically. Qualitatively McGhee-von Hipple theory predicts that at low total dye concentration, the concentration of bound dyes and, consequently, the degree of twist will initially increase from zero as a function of total dye concentration. As the total dye concentration became greater, the concentration of bound dyes and the degree of twist saturates. This qualitative behavior is exhibited by the data presented in Fig. 3. With sufficiently precise data the constants *α, K*, and *n* can be extracted. The values of these parameters are, however, sensitive to the curvature of the knee where the concentration of bound dyes levels off as a function of the total dye concentration. We found that the scatter in our data was too large to extract meaningful values for these three constants. The curves shown in Fig. 3, are examples obtained from the McGhee-von Hipple theory. However they should be regarded as guides to the eye since curves with greatly differing values for *α, K*, and *n* would give similar results. The values of the fitting parameters used here to obtain these curves are given in the Supporting Information S6.

It should be noted that the error bars in Fig. 3 represent the standard deviation of the data from the mean, and not the uncertainty of the mean. Since each data point represents the average twist of more than 100 nanorails, the statistical uncertainty in the mean is an order of magnitude smaller. The standard deviation of the data from the mean can provide information on the physical processes responsible for the stochastic behavior. An estimate for the standard deviation of *N*_*T*_, due to statistical fluctuations in the number of bound dyes, can be obtained in the following manner: at complete saturation the number of dyes bound to the nanorail is given by *N/n* where *N* is the number of base pairs in the nanorail (here *N* = 14280). We assume fluctuations in the number of dyes bound to the nanorail to be greatest at about half of the saturation coverage, *N/2n*, and that the standard deviation will be the square-root of this quantity. Hence, one expects the ratio of the standard deviation, Δ*N*_*T*_, in the number of twists, *N*_*T*_, to be *ΔN*_*T*_ */N*_*T*_ = $\sqrt{2n/N}$. Choosing a reasonable value for *n*, *n* = 4, yields *ΔN*_*T*_ */N*_*T*_ = 0.025 for an upper bound. This is an order of magnitude smaller than the standard deviations exhibited in our data, which, for ethidium bromide is 0.34 and for SYBER Green I is 0.41. Hence, the statistical variation in the number of dye molecules binding to the nanorail does not account for the variance in our data. We also estimated the standard deviation, *ΔN*_*T*_, due to thermal motion, based on the torsional stiffness of duplex DNA (Supporting Information S6), but did not take into account stiffness reduction due to the nicks and crossovers in the nanostructure. The number obtained, *ΔN*_*T*_ = 0.26, which can be regarded as an upper bound, is within a factor of two of the experimentally observed values 0.34 and 0.41 for ethidium bromide and SYBER Green I respectively. Hence we conclude that the standard deviation of the twist is likely due to thermal motion. By inserting bases to the original design of the nanorail, we also simulated the twisting of the nanorail using the CanDo^35^ simulation tool. The number of predicted twists increased as the number of inserted bases increased (Supporting Information S7), suggesting that the addition of intercalator dyes increased the space between bases. Furthermore, we studied reversibility of nanorail twisting by removing the intercalator dyes. For example, nanorails originally mixed with 20 µM of SYBR Green I twisted an average 2.74 turns, and after removal of the dyes using dialysis, the number of twists decreased to 1.74 turns (Supporting Information S8).

## Discussion

Global twisting of DNA origami nanorails can be induced by intercalators such as ethidium bromide and SYBR Green I, and the number of twists increases as the concentration of the intercalator increases in solution. We found that reversible post-synthesis conformational changes to the DNA nanostructures is possible through utilization of intercalating agents, the degree of change is dependent on the concentration of the chemical agents and saturates at a certain concentration. The root-mean-square spread in the twist was found to be consistent with the equilibrium fluctuations expected based on the stiffness of duplex DNA. Our nanorail system provides a convenient bed with which to study mechanical distortion of DNA due to DNA binding molecules. We suggest that the effect of the intercalator dyes on the stability of DNA nanostructures – e.g. in presence of enzymes or environments that affects DNA content – should be explored. Chromatin analogous gene expression (CAGE)^21^ structures are potential models. Finally, the current study provides biology with a framework to discuss the interaction of intercalating agents with complex DNA systems at high-level organizations such as chromosomal and sub-chromosomal arrangements.

## Methods

Two 6HB subunits of the nanorail were synthesized independently by mixing 1 pmol of single-stranded M13mp18 DNA and 10 pmol of each individual staple strands, including the nanorail linking crossover strands, in a 100 µl 1× TAEM buffer (1× TAE, 40 mM MgCl_2_; pH 7.5), heating the mixture to 90 °C for 1 min, and reducing the temperature to 20 °C over a course of 60 hours. The produced 6HBs (6HB_A_ and 6HB_B_) were purified using Amicon Ultra-0.5 mL centrifugal 100 K filters [MilliporeSigma]. Formation of the nanorail was induced by mixing equal amounts of purified 6HB_A_ and 6HB_B_ and incubation for 60–480 min at 50 °C. All upstream experiments were conducted in a final nanorail concentration of 1.4 nM.

Twist of the structures were induced by addition of concentrated intercalator dye to the assembled origami solutions and left at room temperature for two hours before the application of the sample to the mica surfaces. To further study the reversibility of nanorails twisting, we removed the intercalating agents by dialyzing 75 µl twisted nanorails (at 20 µM intercalator concentration) for 24 hours in 500 mL 1× TAEM buffer using a 20 K Slide-A-Lyzer dialysis unit [Thermo Fisher Scientific].

Non-contact tapping AFM mode was preferred mode when collecting images of the nanorails. Non-contact tapping mode utilized low tip oscillations, around 1 to 5 nm, and attractive forces from the sample by tuning the probe to frequencies above the peak resonant frequency. Low tip oscillations and attractive forces reduces tip degradation, allowing for higher resolution AFM images. Collected AFM images were statistically analyzed, where generally a large number of nanorails were counted to create means and variance for each concentration examined. We then used ScanAsys^TM^ AFM mode, which incorporated Peak Force Tapping mode with automatic image optimization, to image the nanorails chirality.

## Electronic supplementary material

Supporting Information
