# Supplementary material for: Twisting of DNA Origami from Intercalators
Source: Sci Rep. 2017 Aug 7;7:7382. doi: 10.1038/s41598-017-07796-3 (PMC5547094; doi:10.1038/s41598-017-07796-3)

Supporting Information

Twisting of DNA Origami from Intercalators

Reza M. Zadegan[1], Elias G. Lindau[1], William P. Klein[1], Christopher Green[1], Elton Graugnard[1], Bernard Yurke[2], Wan Kuang[2], & William L. Hughes[1]

Table of contents

**S1.** Design of nanorail	1
**S2.** Twist chirality	1
**S3.** AFM imaging and analysis	2
**S4.** Concentration depended twists	2
**S5.** qPCR	2
**S6.** Calculations	4
**S7.** CanDo simulations	5
**S8.** Reversibility of the twist	5
**References**	6
**Appendix:** AFM images	7

[1]Micron School of Materials Science and Engineering, Boise State University, Boise, Idaho, United States 83725,
[2]Department of Electrical & Computer Engineering, Boise State University, Boise, Idaho, United States 83725.
Correspondence and requests for materials should be addressed to W.L.H. (email: willhughes@boisestate.edu)

S1. Design of nanorail

Nanorail was designed using in house developed software, in combination with caDNAno[1], and CanDo[2]. All of the designs were based on a six-helix DNA origami nanotube described elsewhere[3]. The M13mp18 scaffold layout is shown in fig. S1a as blue lines. Only representative cross linking segment of the nanorail is shown in fig. S1a. The staple strands (colored lines) are arranged into 86 columns and numbered from left to right. The nanorail consisted of two different 6HBs ($6HB_A$ and $6HB_B$) that were synthesized separately and designed to have a total of 18 cross-linking strands at nine locations along the two 6HBs. In fig. S1b the cross-linking strands are shown in red and green. A two-dimensional and three-dimensional cross-sections of the nanorail are shown in fig. S1b and S1c, respectively. For list of strands and sequences refer to ref 3.

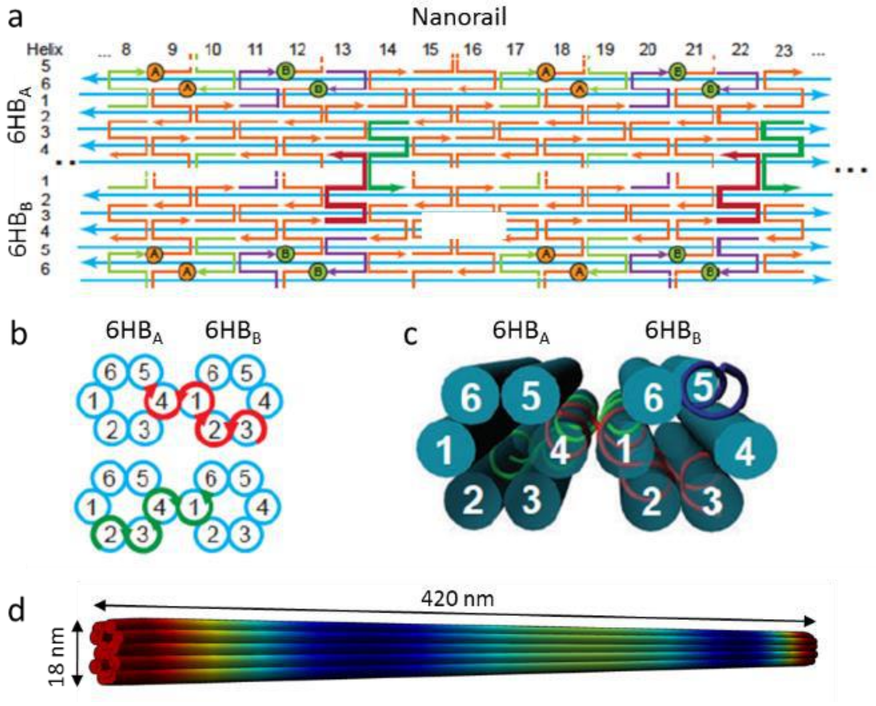

Figure S1. Schematic design of the nanorail. (a) Illustration of one segment of nanorail, in-which the two 6HBs are cross-linked via strands shown in dark green and red. (b) The routing of the linking crossovers between the two 6HBs. Blue circles represent the helices and green and red arrows represent the linking strands. (c) 3D model of the crosslinked 6HBs that results in formation of a nanorail. Helices are shown by turquoise cylinders and cross-over strands are shown as thinner colored lines. (d) Analysis of the structure using CanDo[2]. The red areas of the model represent the more flexible parts of the structure whereas blue regions are stiffer.

S2. Twist chirality

Characterization was done to investigate the chirality of the nanorails once twisted. Chirality can be observed by the direction of the crossing of both 6HBs relative to each other. ScanAsys™ AFM mode was the chosen method to image the nanorails chirality; which incorporates Peak Force Tapping mode with automatic image optimization. Fig. S2 shows AFM images of scans that were run at angles of 0°, 45°, 90°, and 135°. We recorded both trace and retrace images to obtain 360 degrees of scanning. Scanning 360° allows for eliminating the effect that the probe geometry induces on imaging of the nanorail. The trace and retrace images show slight differences in both 6HBs when they are crossing paths. The

right 6HB shows continual height changes during a half twist and the left 6HB shows an abrupt change once the half twist occurs, this alludes to having left-handed chirality. The chirality of the nanorail could also be visualized using the adhesion channels of each scanned angle, in which the nanorail identified to have left-handed chirality.

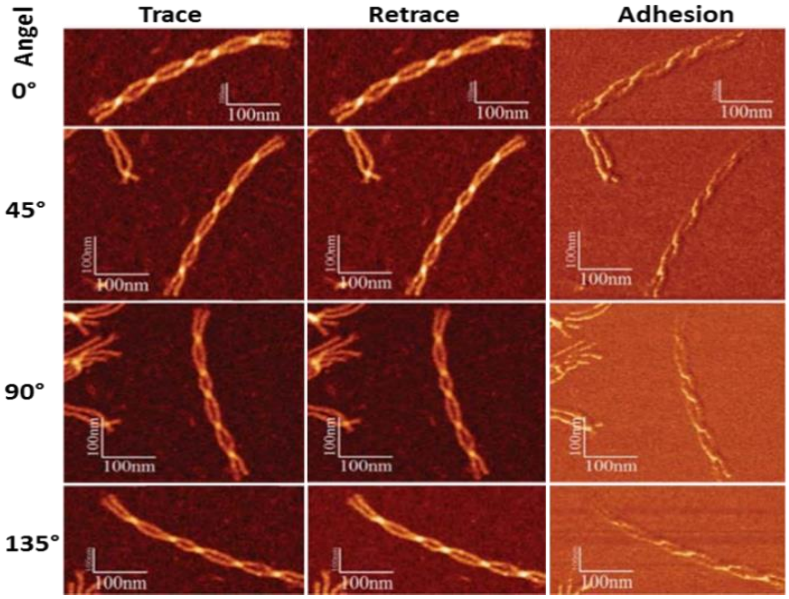

Figure S2: *AFM images used to characterize the chirality of the nanorail. AFM images of scanning angles 0, 45, 90, and 135 degrees were utilized to investigate the chirality of the nanorail once twisted.*

S3. AFM imaging and analysis

The nanorails described in the paper were imaged using AFM. Non-contact tapping mode was the select method when collecting images of the nanorails. Non-contact tapping mode utilized low tip oscillations (around 1 to 5 nm) and attractive forces from the sample by tuning the probe to frequencies above the peak resonant frequency. Low tip oscillations and attractive forces reduces tip degradation allowing for higher resolution AFM images. Collected AFM images were statistically analyzed, where generally a large number of nanorails were counted. Fig. S3 shows an example of analyzed AFM images where the images were re-colored to black and white to facilitate visual tracking of individual nanorails. Both ethidium bromide and SYBR green show similar distributions of twisted nanorails.

S4. Concentration depended twists

We further analyzed the distribution of twists (fig. S4-1), and calculated the statistical mean and standard deviations for all samples (fig. S4-2). There are a few possible reasons for the increase of experimental error; (i) AFM imaging of 3D objects could affect the properties of the objects resulting in more observed error, and (ii) another possible reason is that the sample-mica interaction could affect the amount of nanorail twisting.

S5. qPCR

AFM imaging of the twisted samples when treated at higher concentration of the intercalators (30 μM or more), usually resulted in visualization of bare mica. To investigate whether the high pressure of the intercalation process could cause sheering of the DNA nanostructures, we performed qPCR studies of specific region of the M13mp18 template.

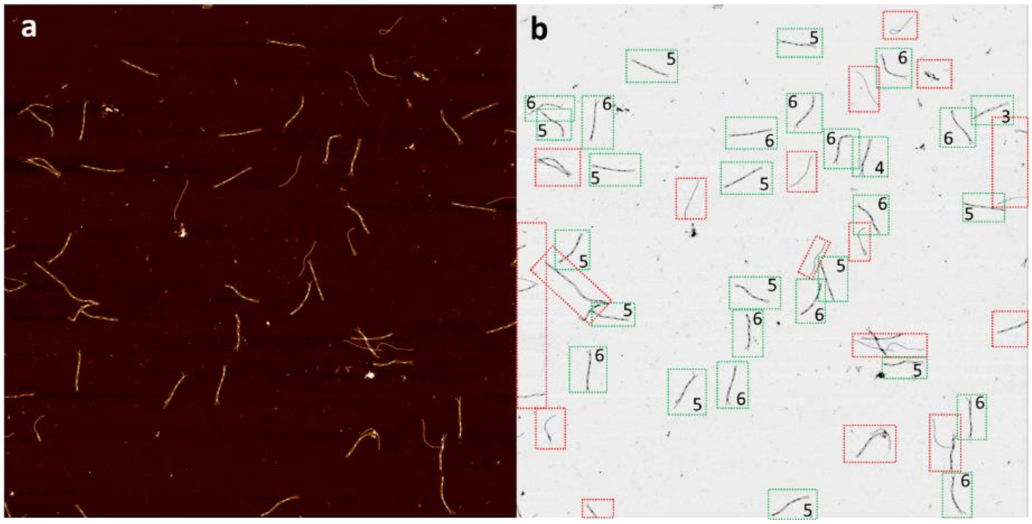

Figure S3. *Example of AFM image (4 µm × 4 µm) of the twisted structures. (a) Original AFM image of the twisted DNA origamis when treated with 20 µM EtBr. (b) Similar image when processed for data analysis. Red squares illustrate the particles that are excluded from quantifications and green squares refer to individual origamis that number of twists could be clearly distinguished. Numbers represent the number of the crosses and represent half twist.*

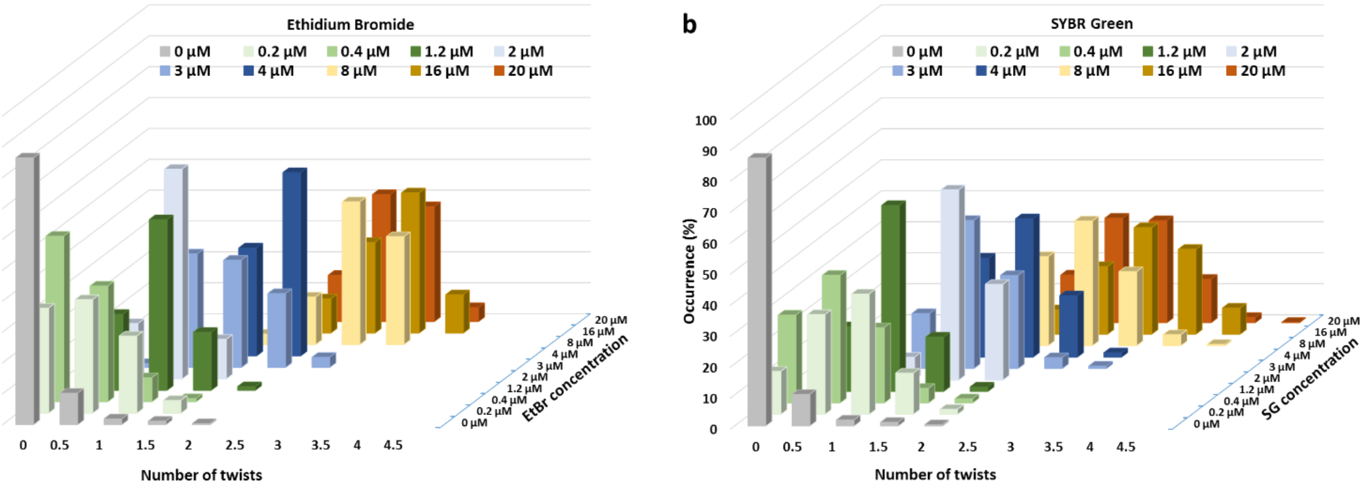

Figure S4-1. *Twisting increased as the [dye] increased from 0 to 20 µM. Summary of twisting effect of intercalators on DNA nanorails for samples treated with EtBr (a) and SG (b).*

A region consisting of 160 bases on the M13mp18 was selected to be amplified and simultaneously a probe in that region was tracked to make sure the quantifications are accurate and specific. A summary of results is shown in fig. S5. The qPCR results indicate that the SYBR Green I dye would affect the integrity of single stranded M13mp18 (ss-M13) more than the nanorails. This could be interpreted as the single stranded DNA is more vulnerable to the sheering effect of intercalator and that the intercalator does not affect the integrity of the majority of the nanorails. Another explanation may be the reduced activity of DNA polymerase in presence of the GYBR green I. The dye is well known to intercalate

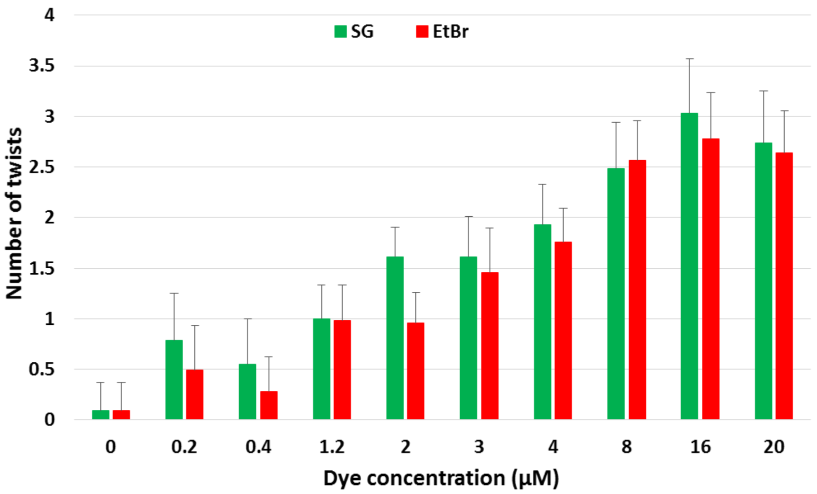

Figure S4-2. *Calculated statistical mean of the nanorails populations. Error bars represent the standard deviations.*

the double stranded DNA, hereby nanorail, and thus one might assume the concentration of dye has been decreased in qPCR mix when exposed to nanorail, and the lower the concentration of free dye in the qPCR mix the higher the activity of the DNA polymerase enzyme. DNA recovery was calculated as $DNA\ recovery\ (\%) = \frac{\text{Conentration of sample} \times 100}{\text{Concentration of initial DNA material}}$.

The following primers and probe were used to conduct the study.
M13-FWD-SeqP01 CGCCAGGGTTTTCCCAGTCACGAC
M13-BWD-SeqP02 AGCGGATAACAATTTCACACAGGA
M13-qPCR-Probe01 /56-FAM/CGGTACCCG/ZEN/GGGATCCTCTAGAGTCGACCTGCAGGCATGC/3IABkFQ/

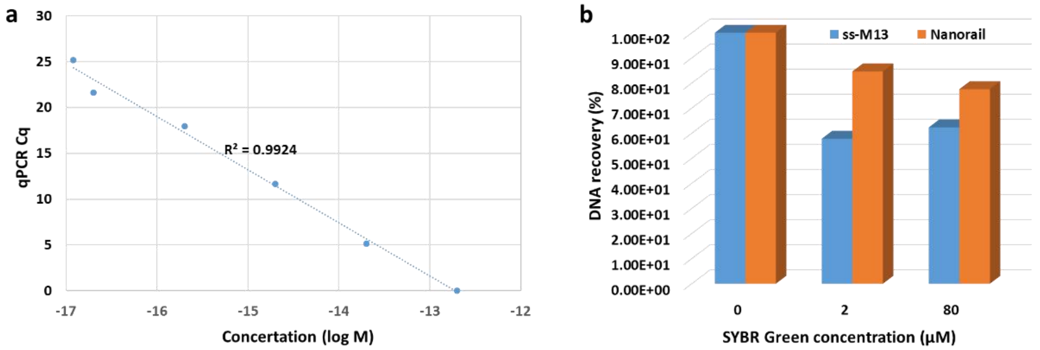

Figure S5. *(a) qPCR standard curve proves our experimental settings are reliable. (b) Quantification of the DNA recovery (in percentage of original material) after treatment with SYBR green dye both in form of single stranded M13 (ss-M13) or in form of nanorails.*

S6. Calculations

The curves of fig. 3 were obtained by fitting the McGhee-von Hipple theory to the data. The values of the fitting parameters extracted from the fits are as follows: K had the values of 5.04E-01 μM^{-1} and 2.73E-02 μM^{-1} for ethidium bromide and SYBR green I,

respectively. n had the values of 4.0 and 18.0 for ethidium bromide and SYBR Green I, respectively. α had the values of 9.42E-04 and 5.63E-03 for ethidium bromide and SYBR, respectively. The reliability of these values were assessed by fitting to simulated data generated from the theory curves of fig. 3 by adding a random number to each theory value for N_T from a Gaussian probability distribution. This resulted to the same standard deviation from zero that our experimentally obtained means had from the theory values. The values of the fitting parameters extracted from, especially K and n, would vary by as much as several orders of magnitude between simulated data sets. Hence, we have extremely low confidence in the values of the fitting parameters reported here.

The variance of twists, ΔN_{Tc}, due to chemical equilibrium of intercalator attachment was found to be concentration dependent. It increased quickly with the intercalator concentration until it reached a peak value at a concentration of 2 µM. ΔN_{Tc} then decreased slowly as the number of bound intercalator reached saturation. It shows that chemical equilibrium statistical mechanics is not the main cause of experimentally observed variance. The variance ΔN_{Tb} in twist angle due to Brownian fluctuation for the DNA nanorail can be estimated as

$$\Delta N_{Tb} = \frac{1}{\pi}\sqrt{\frac{N_{bp}k_B T}{N_s \alpha_T}}$$

where N_s is the total number of duplex strands along the length of the origami nanorail and N_{bp} is the total number of base-pairs of the structure. α_T is the torsion constant for the angular twisting between successive duplex-DNA base pairs and is measured at $\alpha_T = 6 \times 10^{-19}$ J.[4] For origami nanorail consisting of two 6HBs, $Ns = 12$ and $N_{bp} = 1190$, the variance of twist due to Brownian motion is $\Delta N_{Tb} = 0.26$. It is consistent with the experimental observations for origami nanorails for intercalator concentrations lower than 400 nM. However, the observed variance increases to 0.3 with higher intercalator concentrations, which may indicate that intercalated duplex DNA is less rigid than native duplex DNA.

S7. CanDo simulations

We simulated the occurrence of twists, by inserting a base in every few bases in the designed structure. Simulations were performed using CanDo[2]. Fig. S6 summarizes the results.

S8. Reversibility of the twist

We preformed dialysis to show the reversible nature of the nanorail twisting. 75 µL of 20 µM intercalated sample was placed into a dialysis unit which was then soaked into a 500 mL beaker. Both the beaker and the intercalated sample contained 1x TAEM buffer containing 40 mM $MgCl_2$. Dialysis was performed for 24 hours. After the dialysis was completed the sample was extracted and prepared for AFM imaging. AFM showed that dialysis reduced the average number of twists form 2.74 to 1.74 (fig. S7).

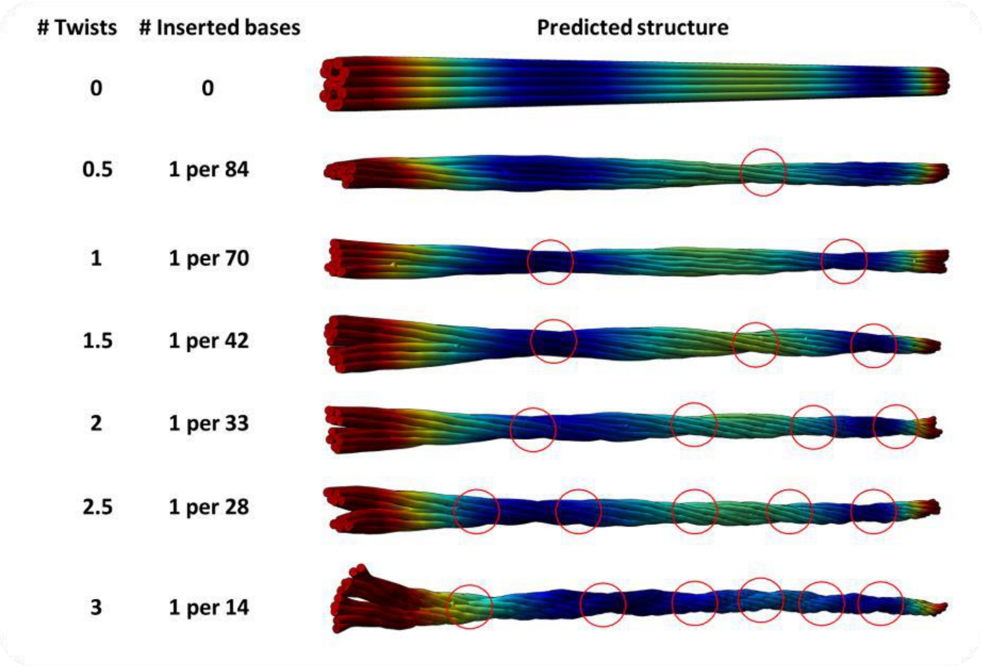

Figure S6. *CanDo simulated twisting of the origami nanorails as a function of number of inserted bases in the structure. One base has been inserted into the original design after each 84, 70, 42, 33, 28 and 14 bases, respectively (top to bottom). The number of predicted twists increases as the number of inserted bases increased. Red circles indicate the points that the two 6HBs cross each other.*

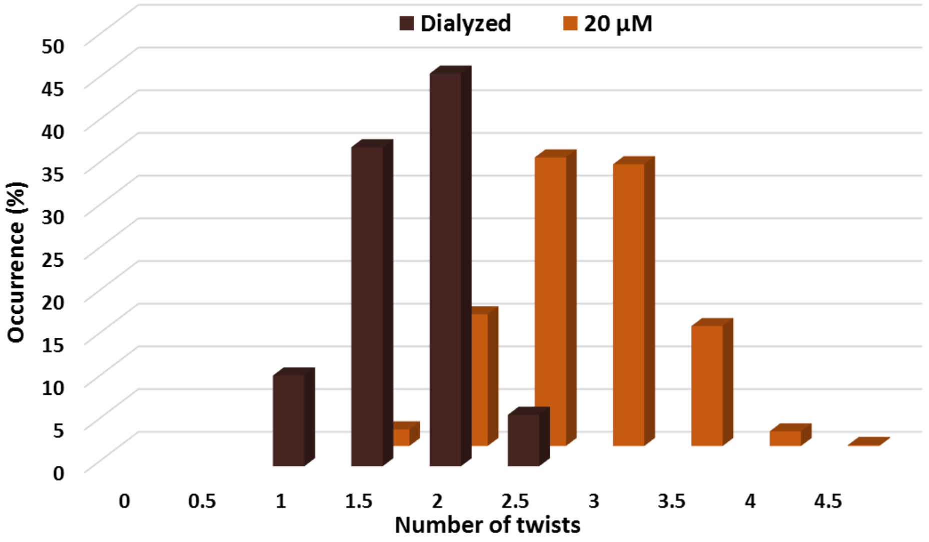

Figure S7. *Reversibility of twist. Twisting decreased as the [SG] decreased from 20 µM during the 24 hours dialysis.*

References

1. Douglas, S. M., Marblestone, A. H., Teerapittayanon, S., Vazquez, A., Church, G. M., *et al. Nucleic Acids Res.* **37,** 5001-5006 (2009).

2. Castro, C. E., Kilchherr, F., Kim, D.-N., Shiao, E. L., Wauer, T., *et al. Nat. Methods* **8,** 221-229 (2011).
3. Klein, W. P., Schmidt, C. N., Rapp, B., Takabayashi, S., Knowlton, W. B., *et al. Nano Lett* **13,** 3850-3856 (2013).
4. Heath, P. J., Clendenning, J. B., Fujimoto, B. S. & Schurr, J. M. *J. Mol. Biol.* **260,** 718-730 (1996).

Appendix: AFM images

The AFM images that have been used for the statistical analysis in this report are provided in the following section; pages i to xlii.

[SYBR Green I (SG)] = 0 µM
[Ethidium Bromide (EtBr)] = 0 µM

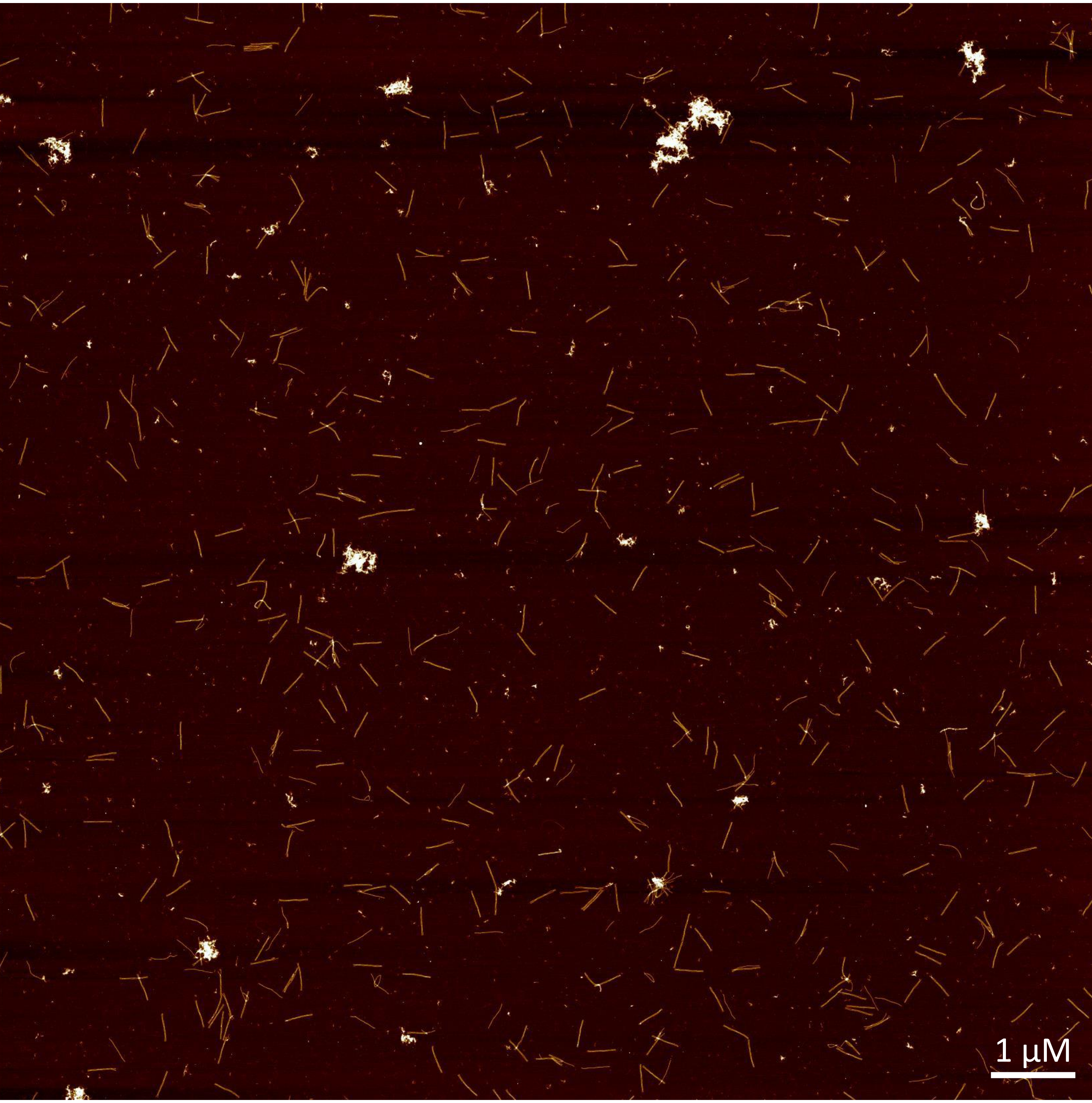

[SYBR Green I (SG)] = 0 μM
[Ethidium Bromide (EtBr)] = 0.2 μM

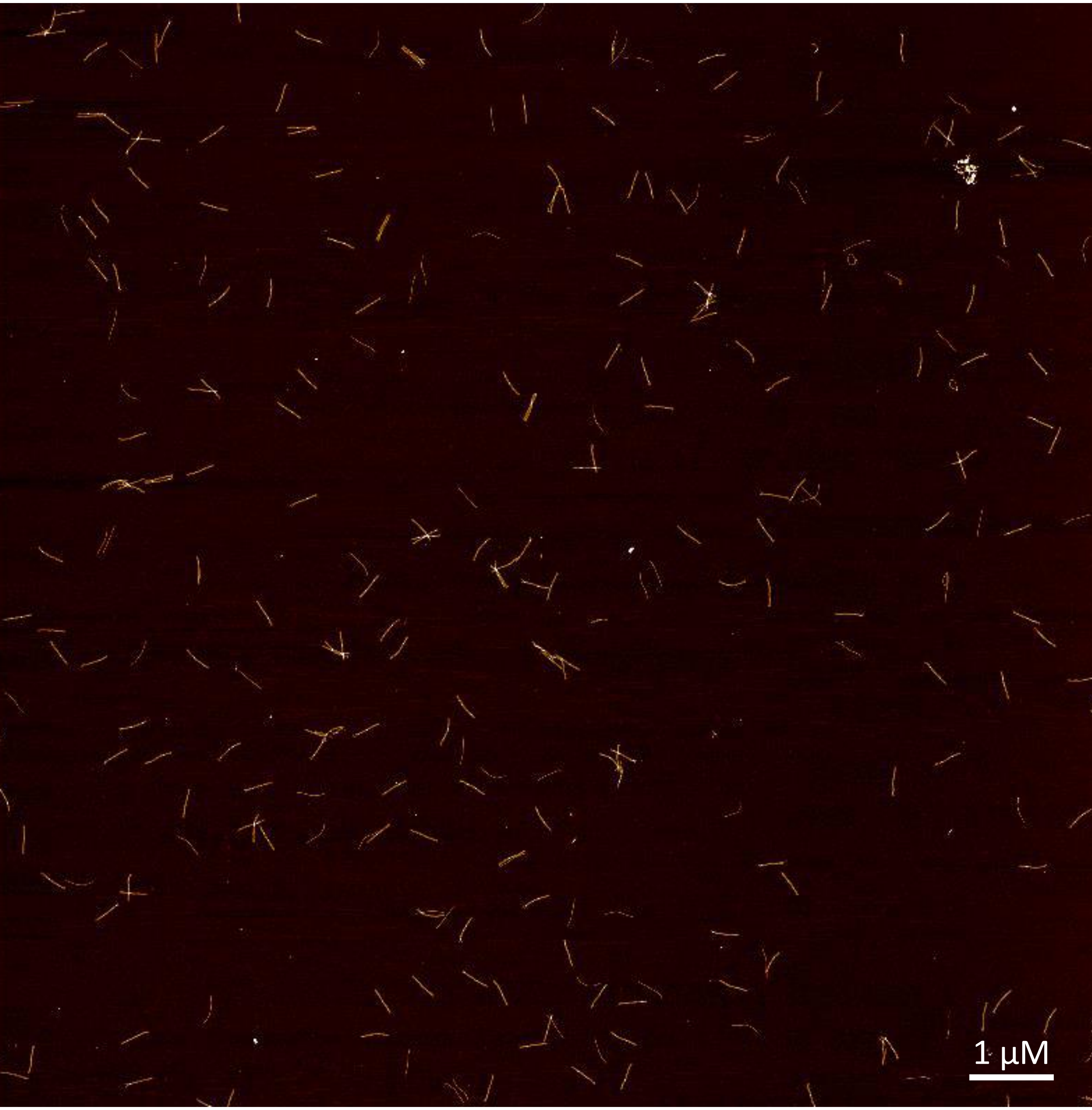

[SYBR Green I (SG)] = 0 µM
[Ethidium Bromide (EtBr)] = 1.2 µM

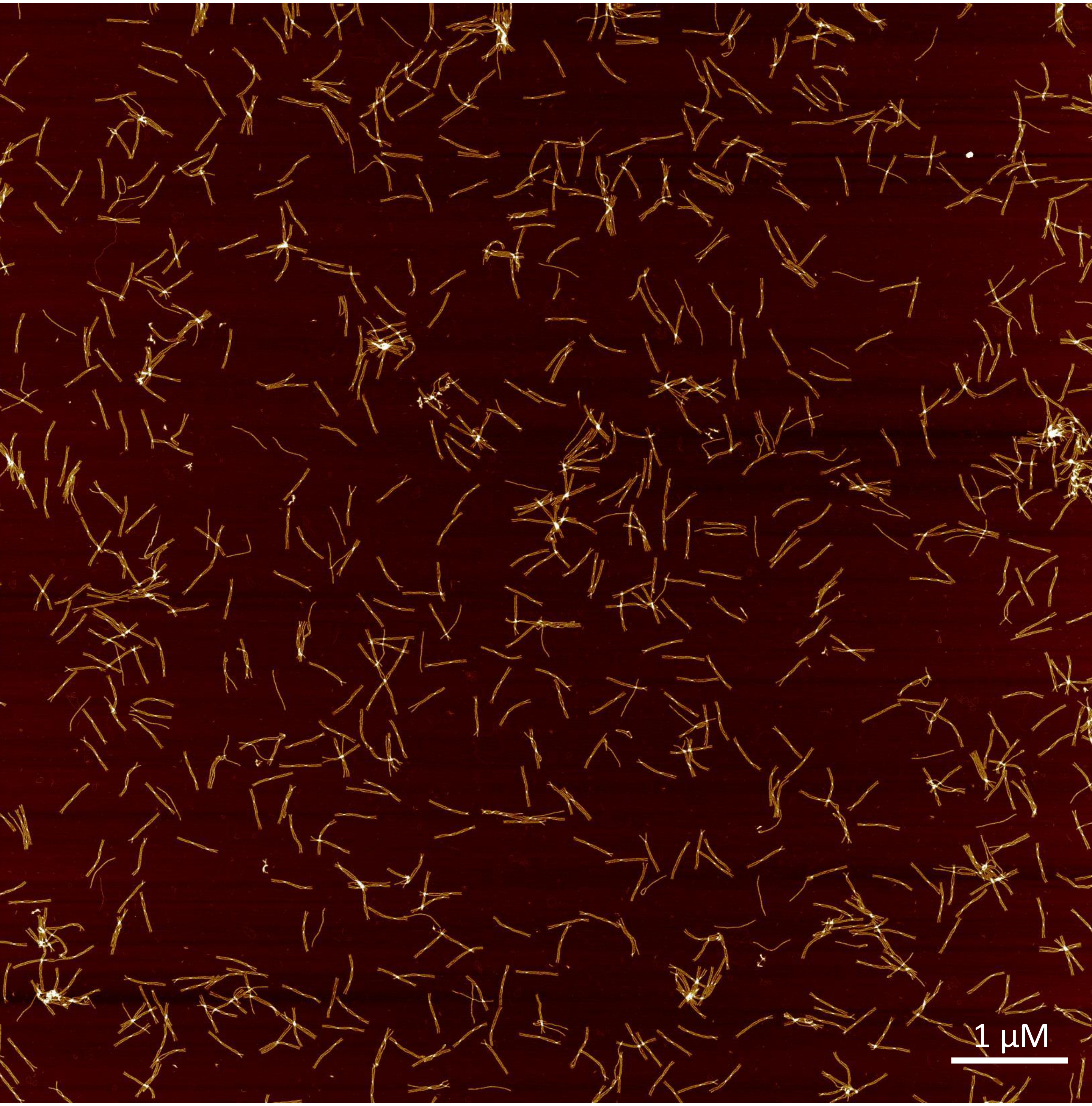

[SYBR Green I (SG)] = 0 µM
[Ethidium Bromide (EtBr)] = 2 µM

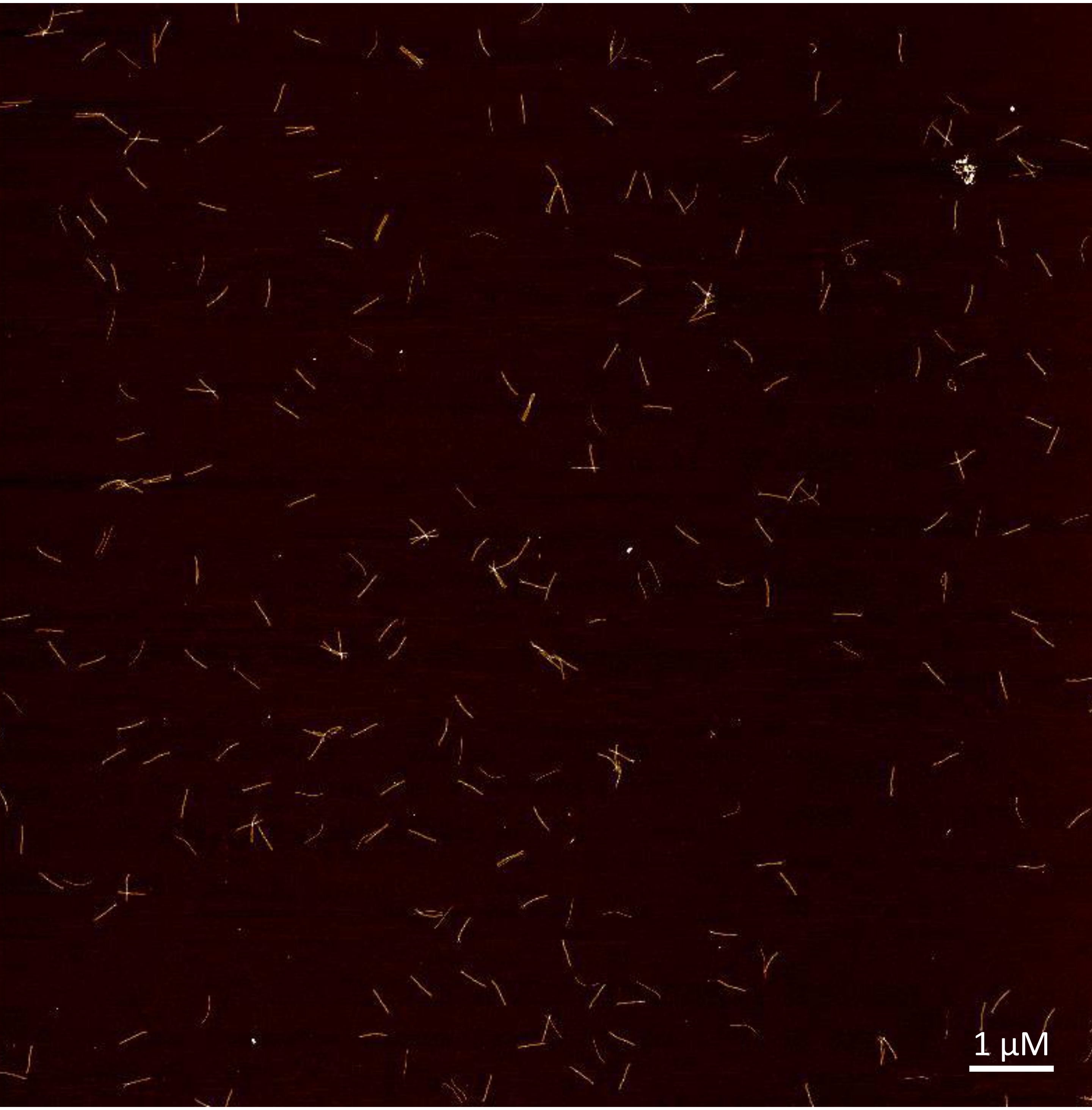

[SYBR Green I (SG)] = 0 µM
[Ethidium Bromide (EtBr)] = 3 µM

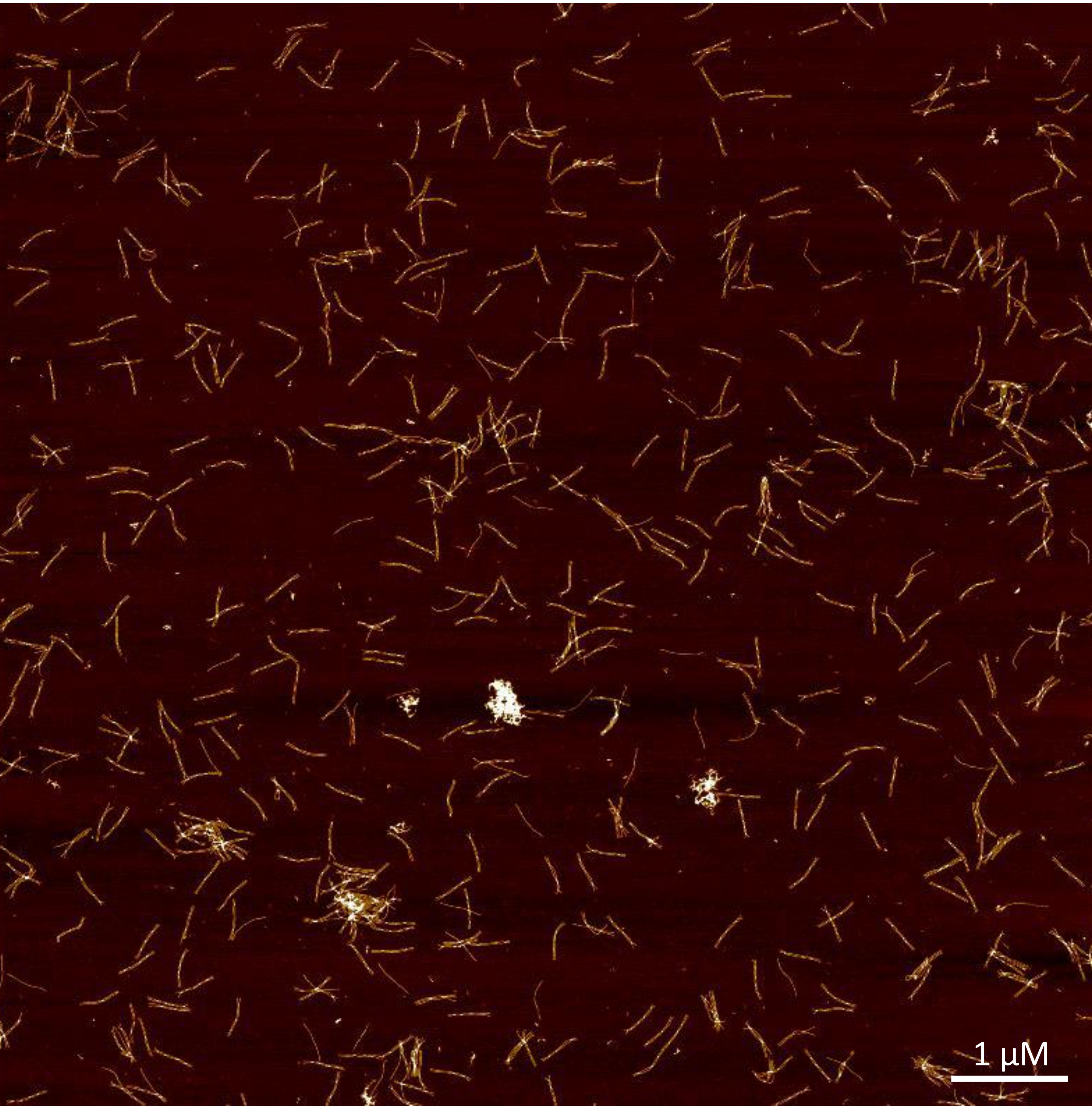

[SYBR Green I (SG)] = 0 μM
[Ethidium Bromide (EtBr)] = 4 μM

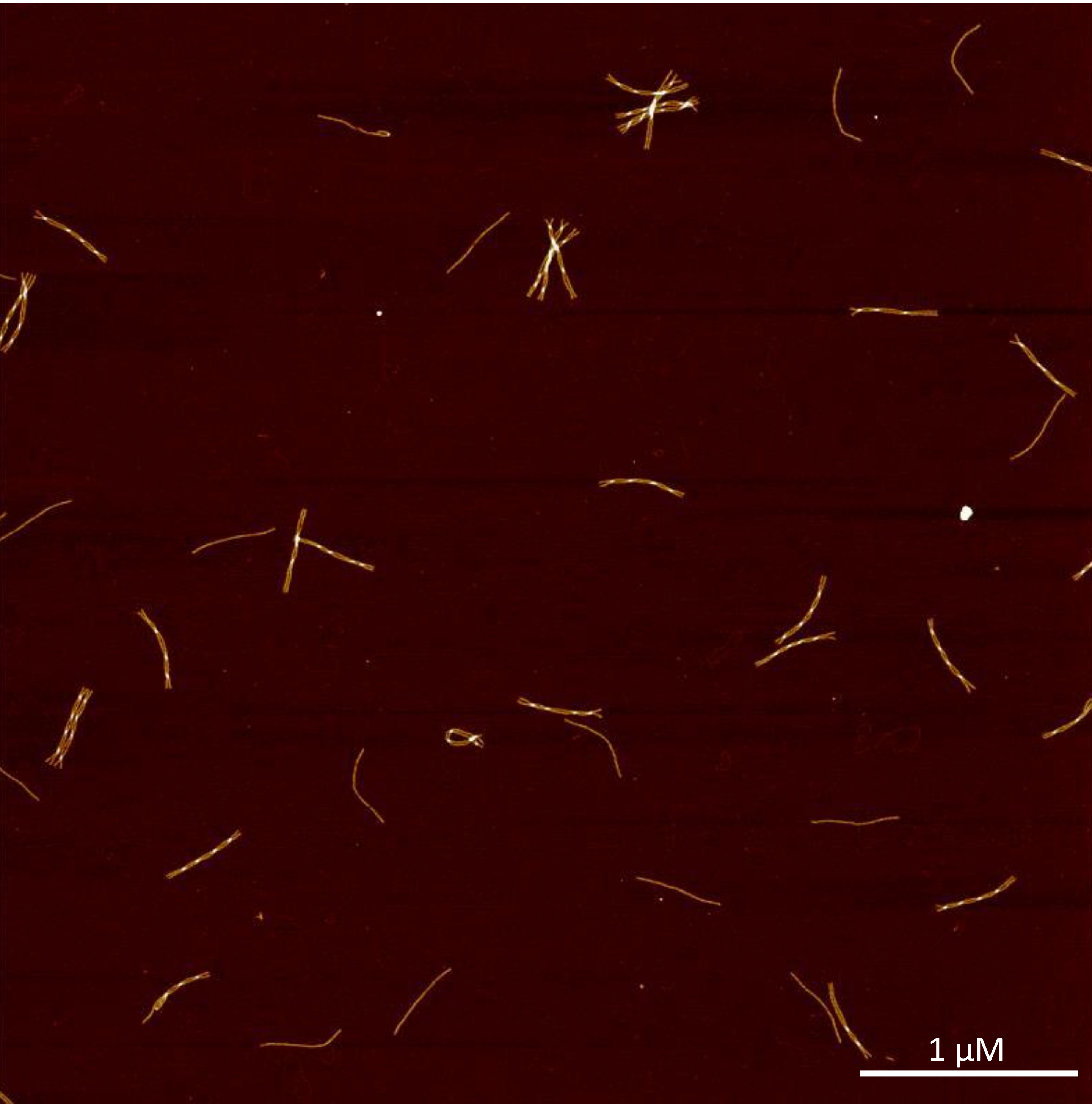

[SYBR Green I (SG)] = 0 µM
[Ethidium Bromide (EtBr)] = 4 µM

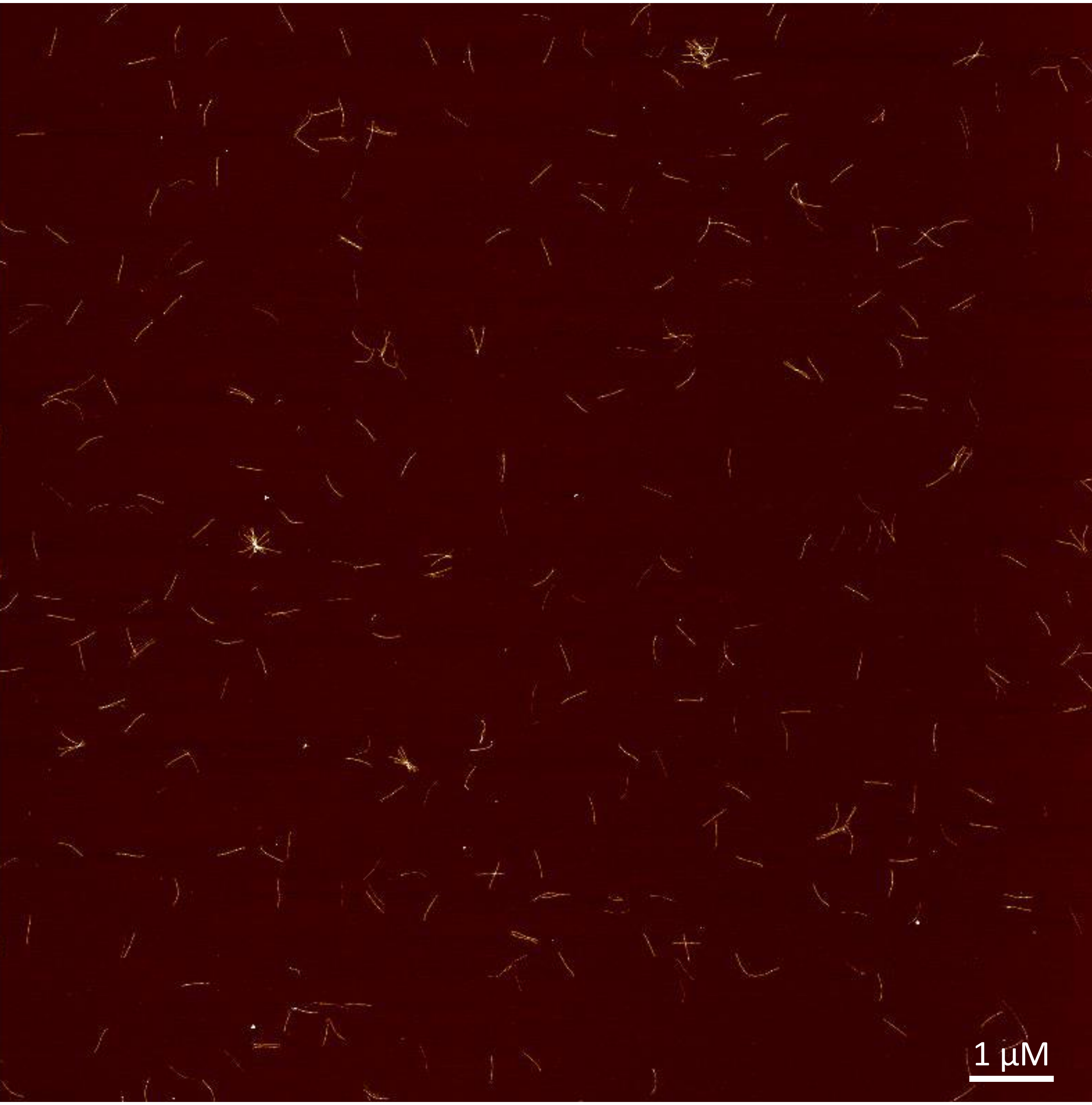

[SYBR Green I (SG)] = 0 μM
[Ethidium Bromide (EtBr)] = 8 μM

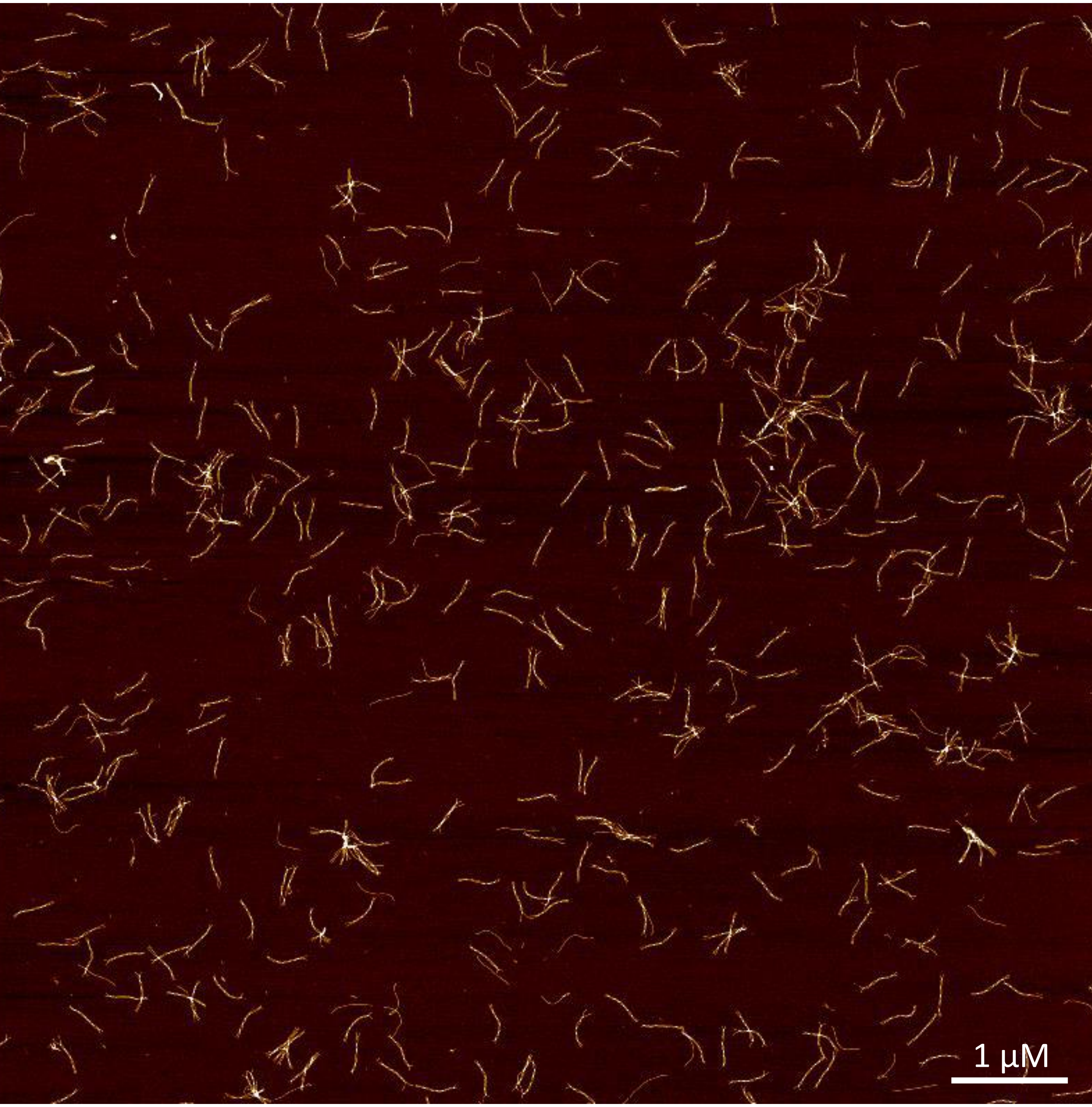

[SYBR Green I (SG)] = 0 μM
[Ethidium Bromide (EtBr)] = 16 μM

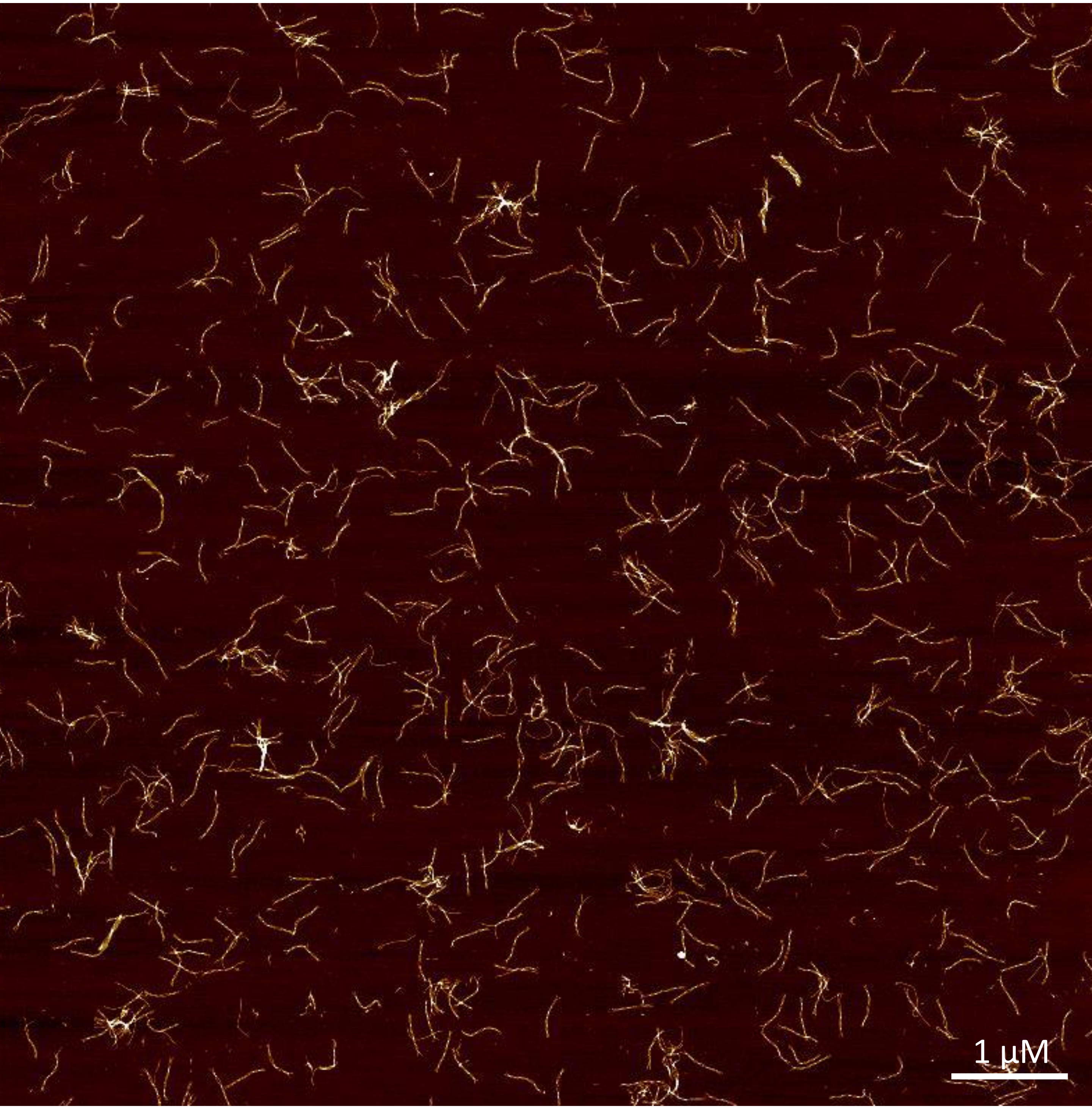

[SYBR Green I (SG)] = 0 µM
[Ethidium Bromide (EtBr)] = 20 µM

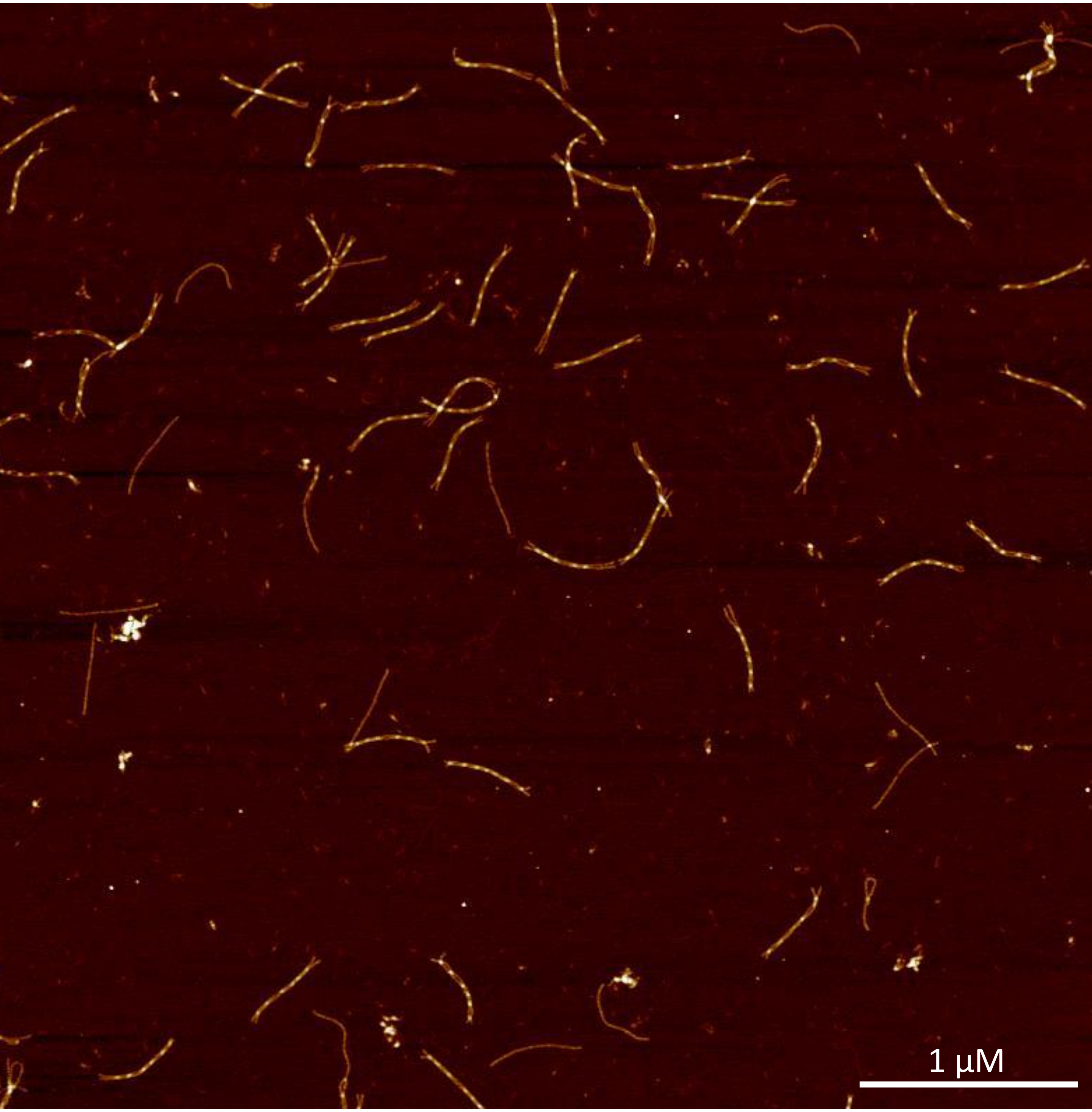

[SYBR Green I (SG)] = 0 µM
[Ethidium Bromide (EtBr)] = 20 µM

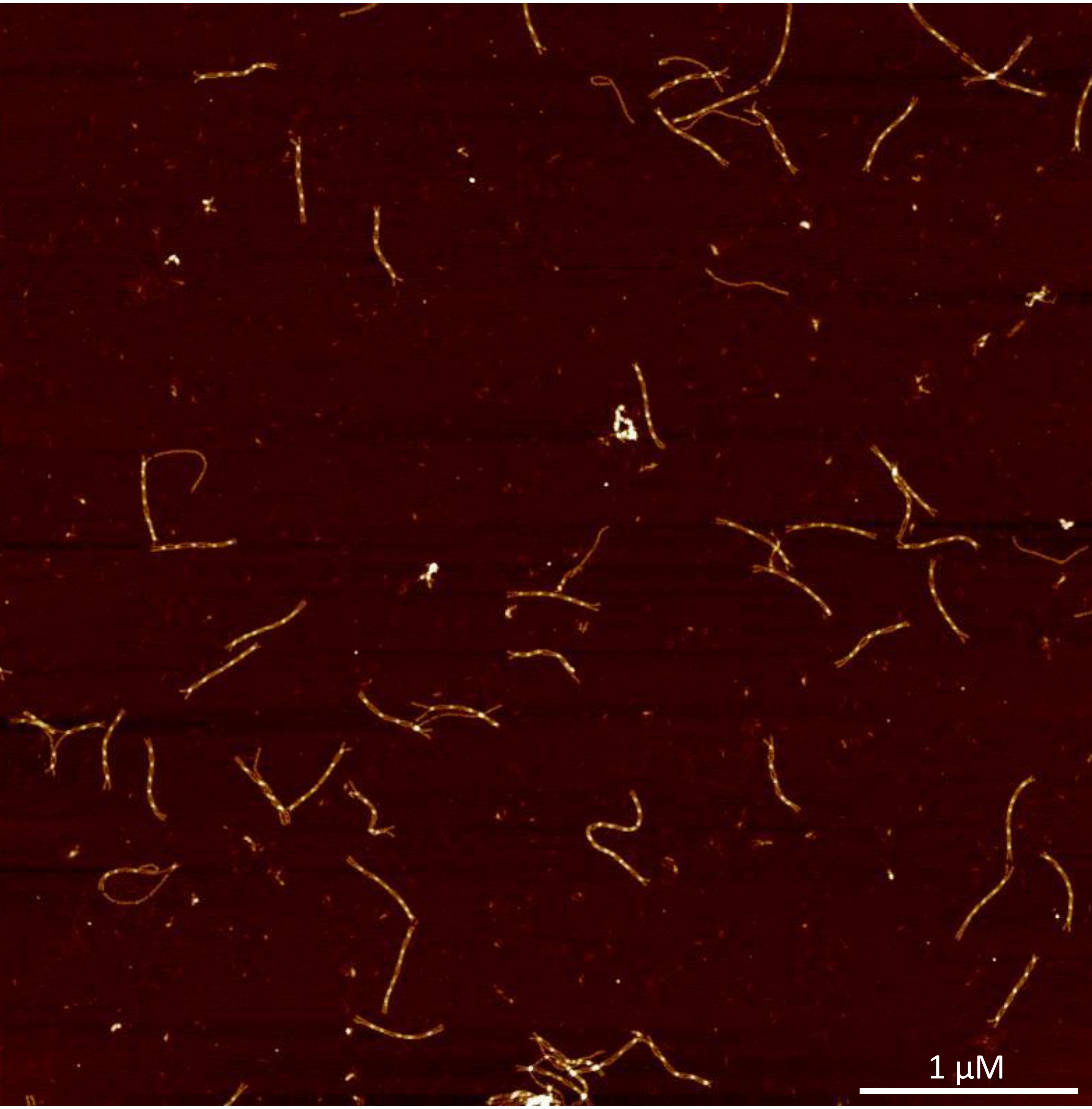

[SYBR Green I (SG)] = 0 μM
[Ethidium Bromide (EtBr)] = 20 μM

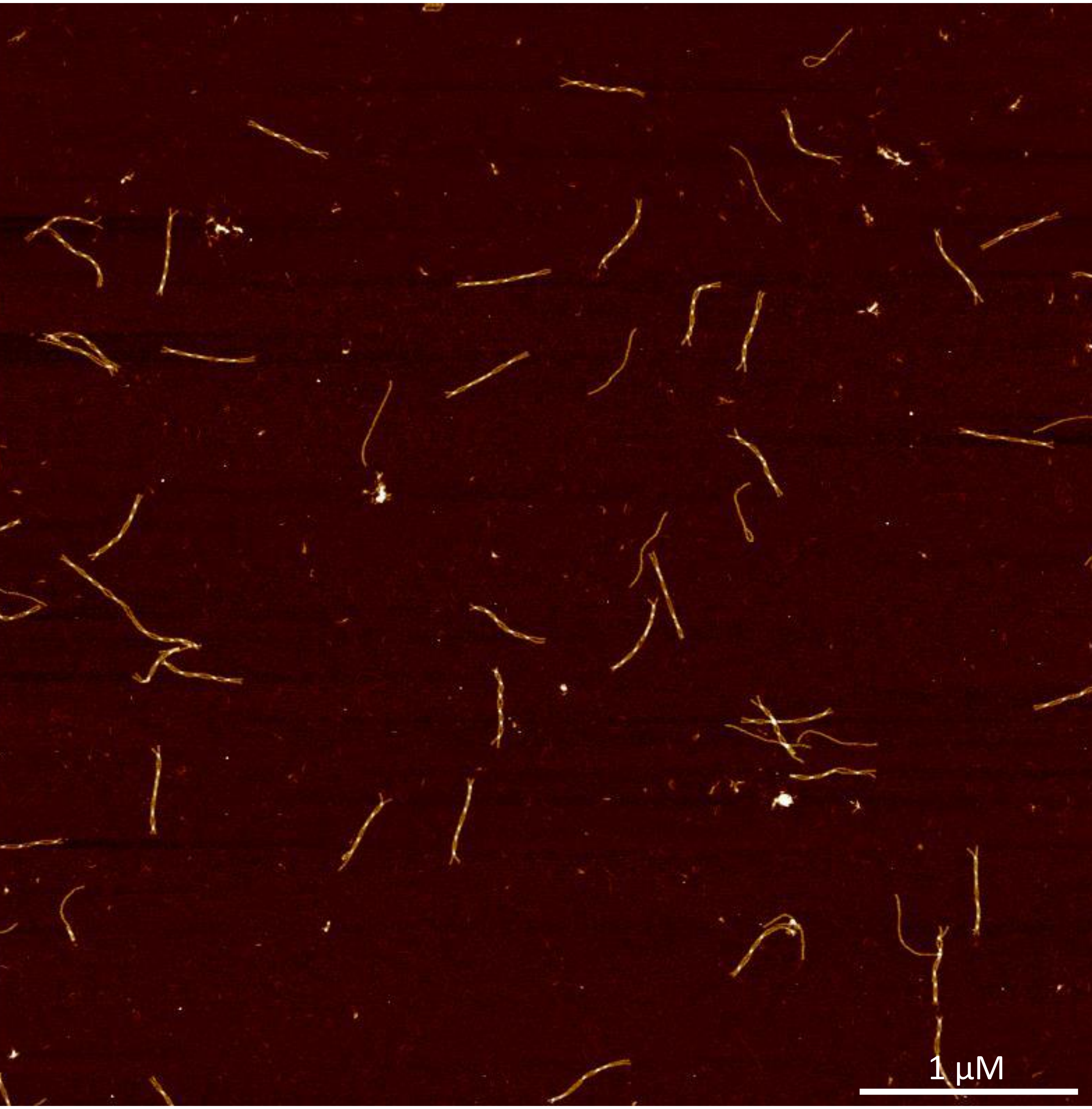

[SYBR Green I (SG)] = 0 μM
[Ethidium Bromide (EtBr)] = 20 μM

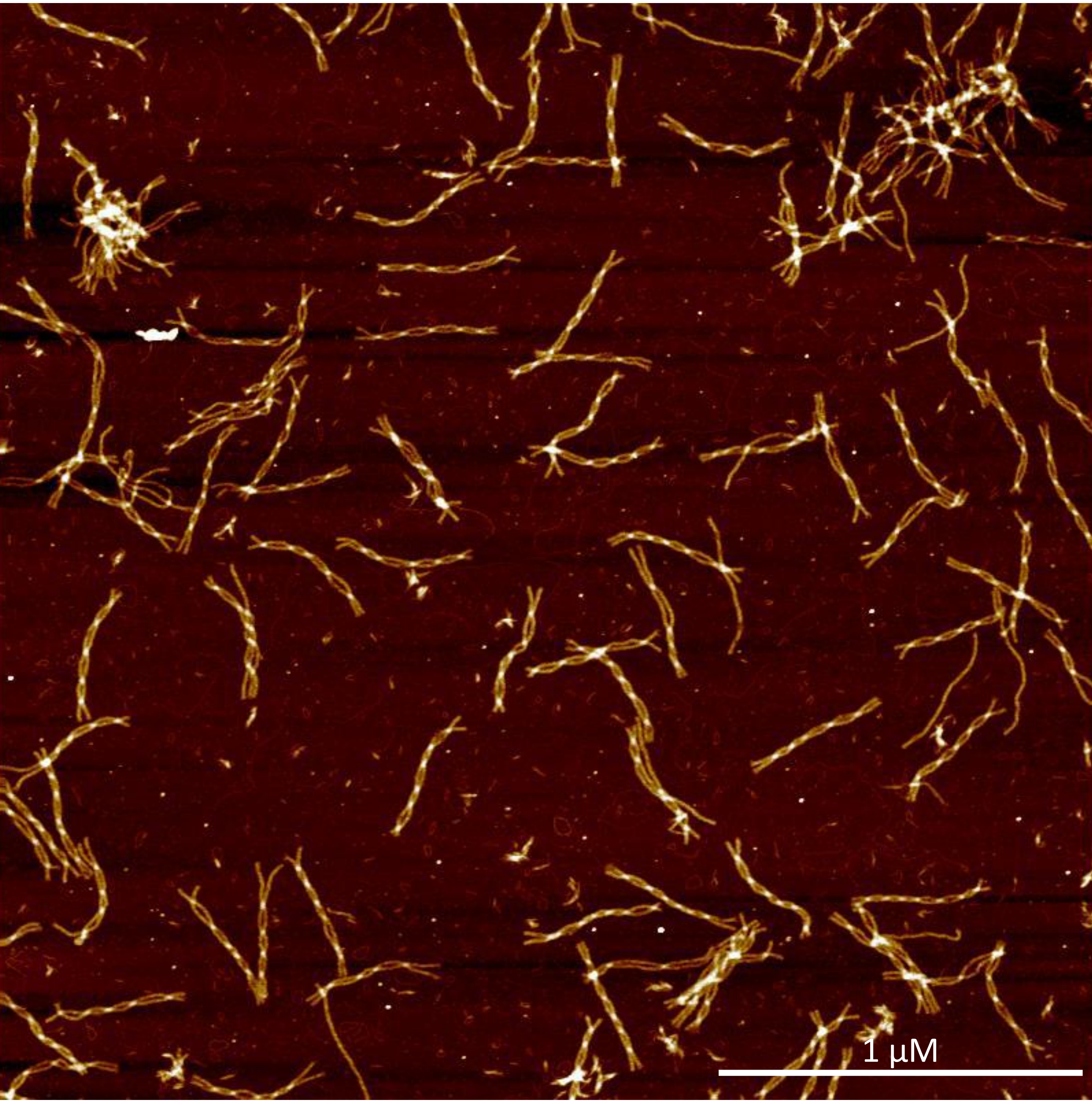

[SYBR Green I (SG)] = 0 µM
[Ethidium Bromide (EtBr)] = 200 µM

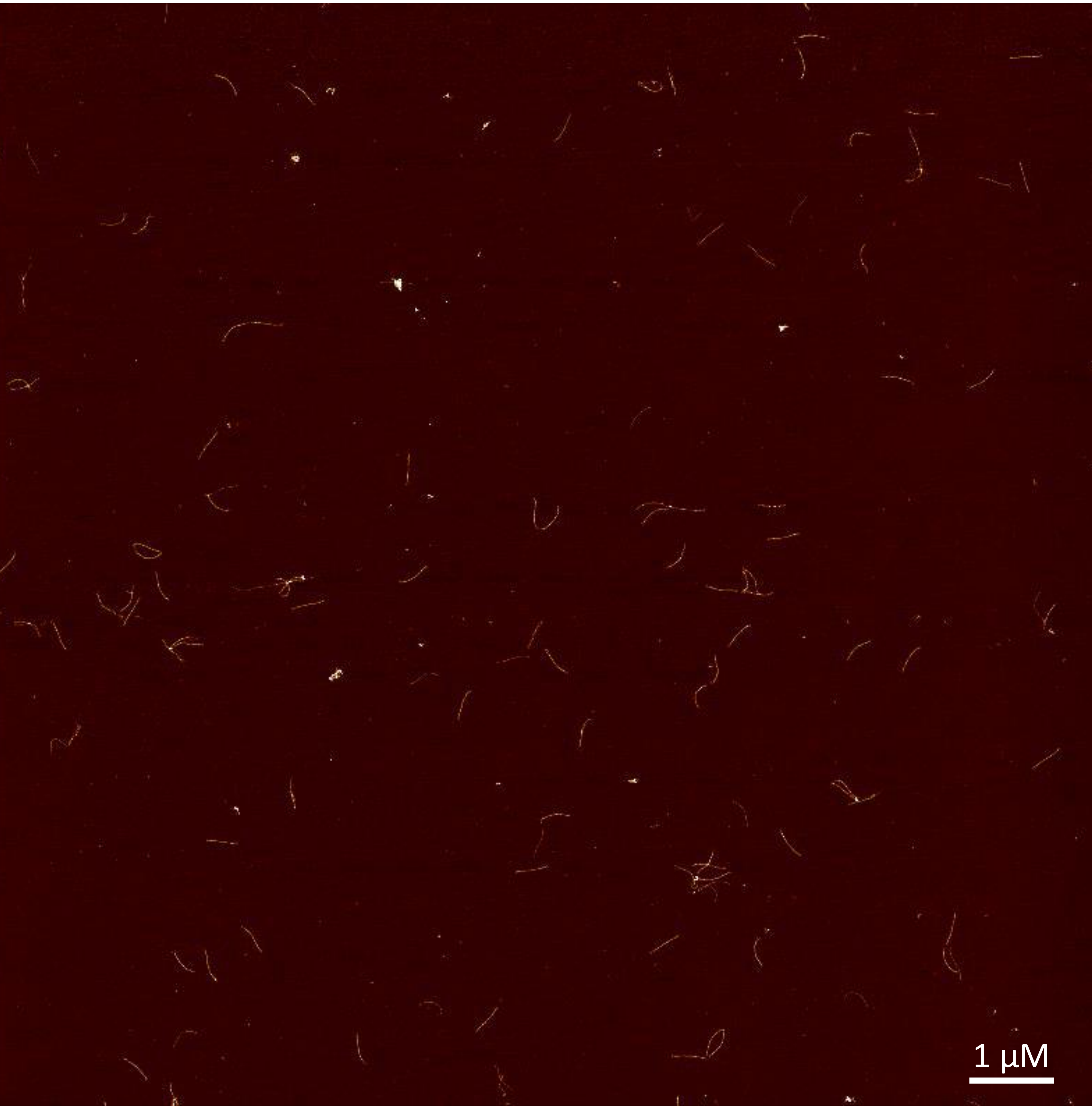

[SYBR Green I (SG)] = 0 µM
[Ethidium Bromide (EtBr)] = 200 µM

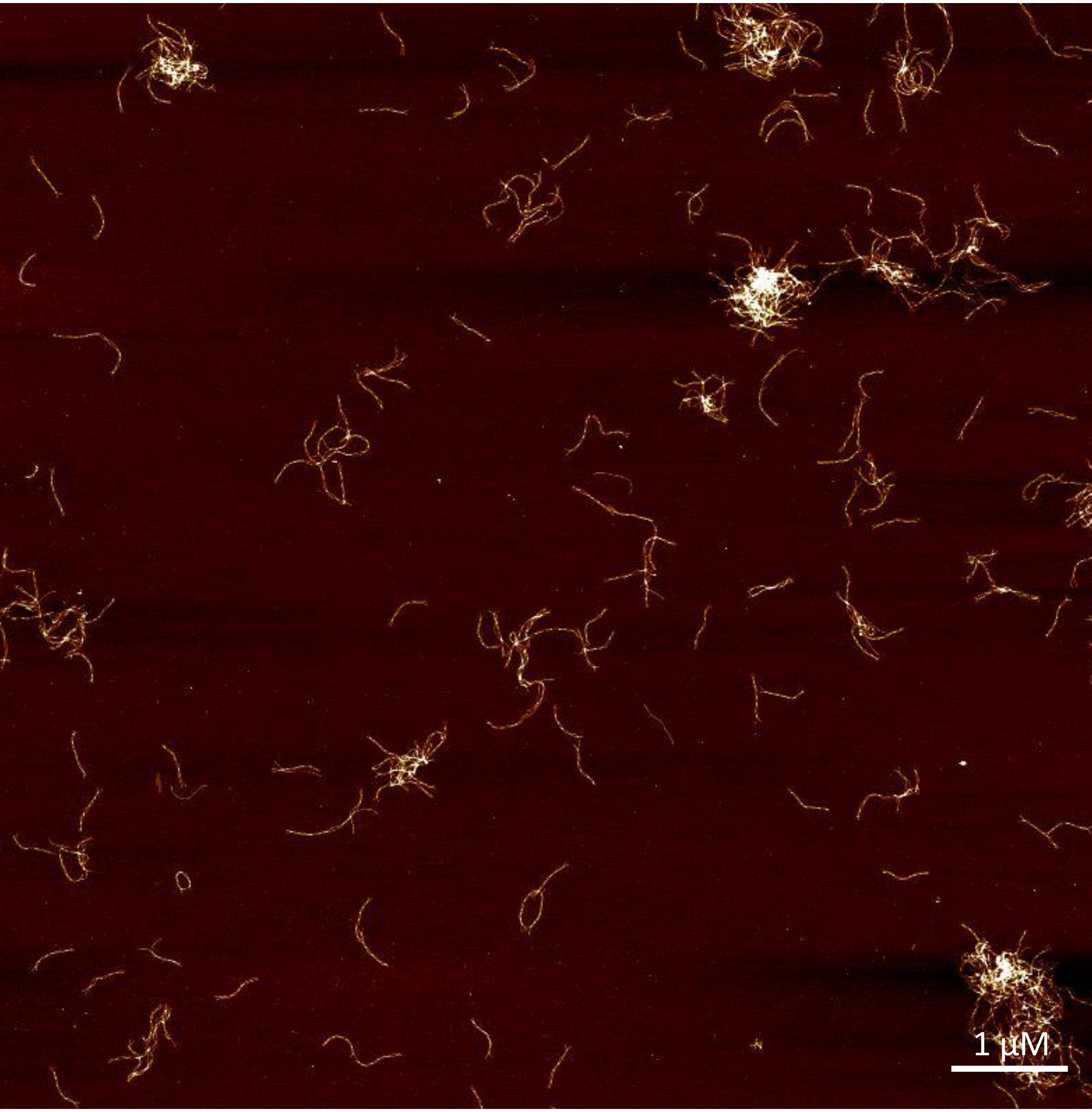

[SYBR Green I (SG)] = 0 µM
[Ethidium Bromide (EtBr)] = 200 µM

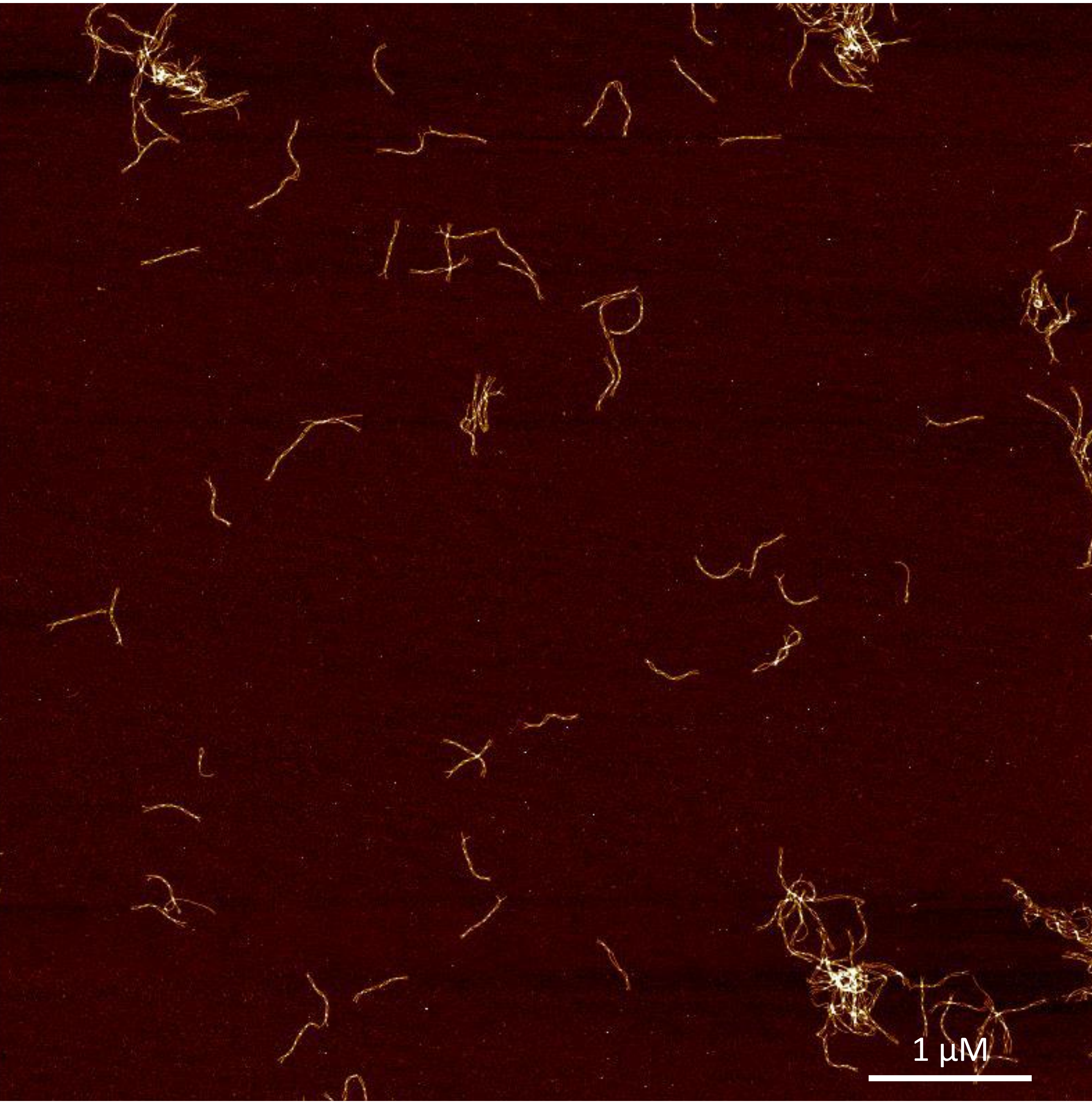

[SYBR Green I (SG)] = 0 μM
[Ethidium Bromide (EtBr)] = 200 μM

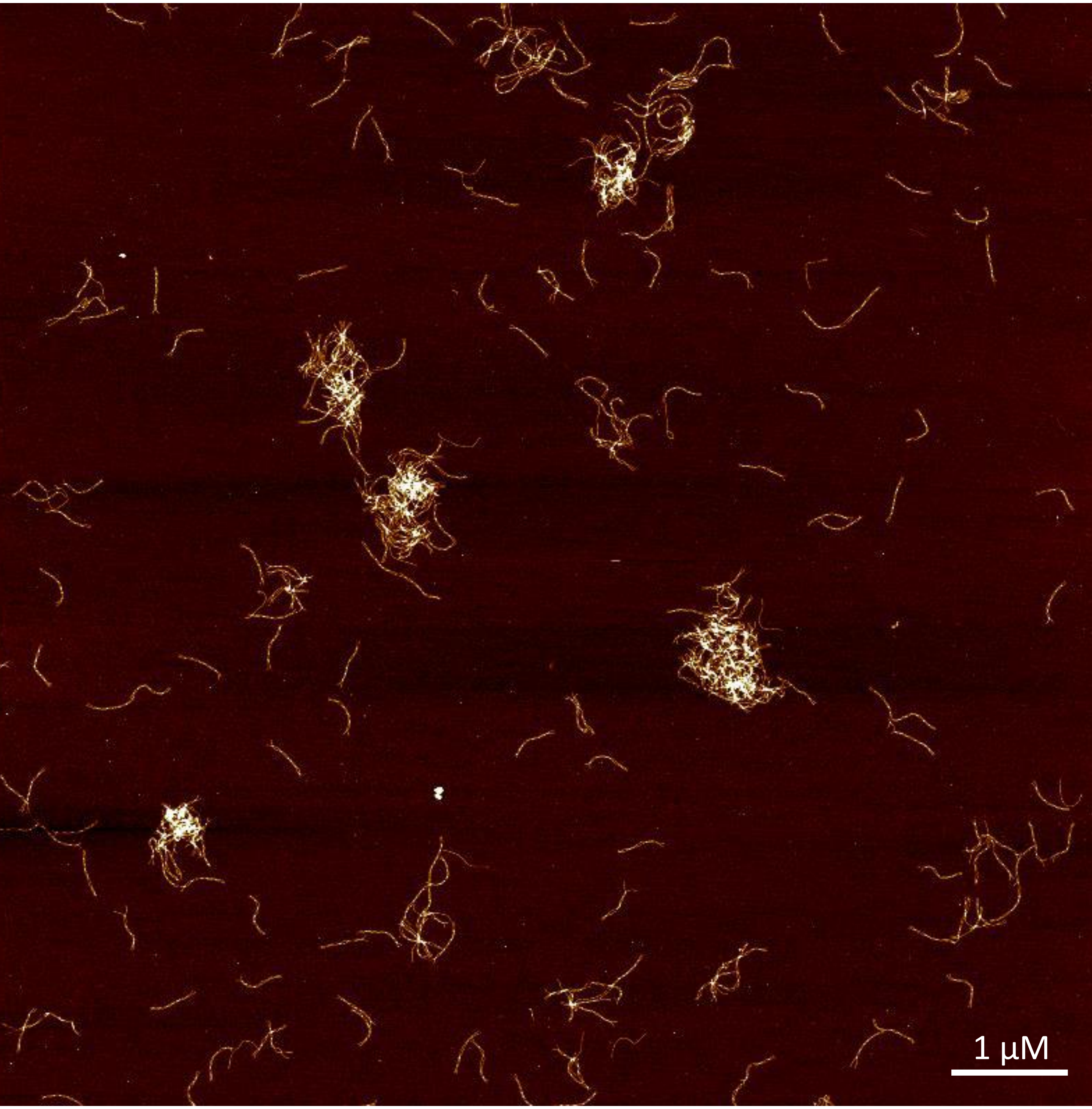

[SYBR Green I (SG)] = 0.2 μM
[Ethidium Bromide (EtBr)] = 0 μM

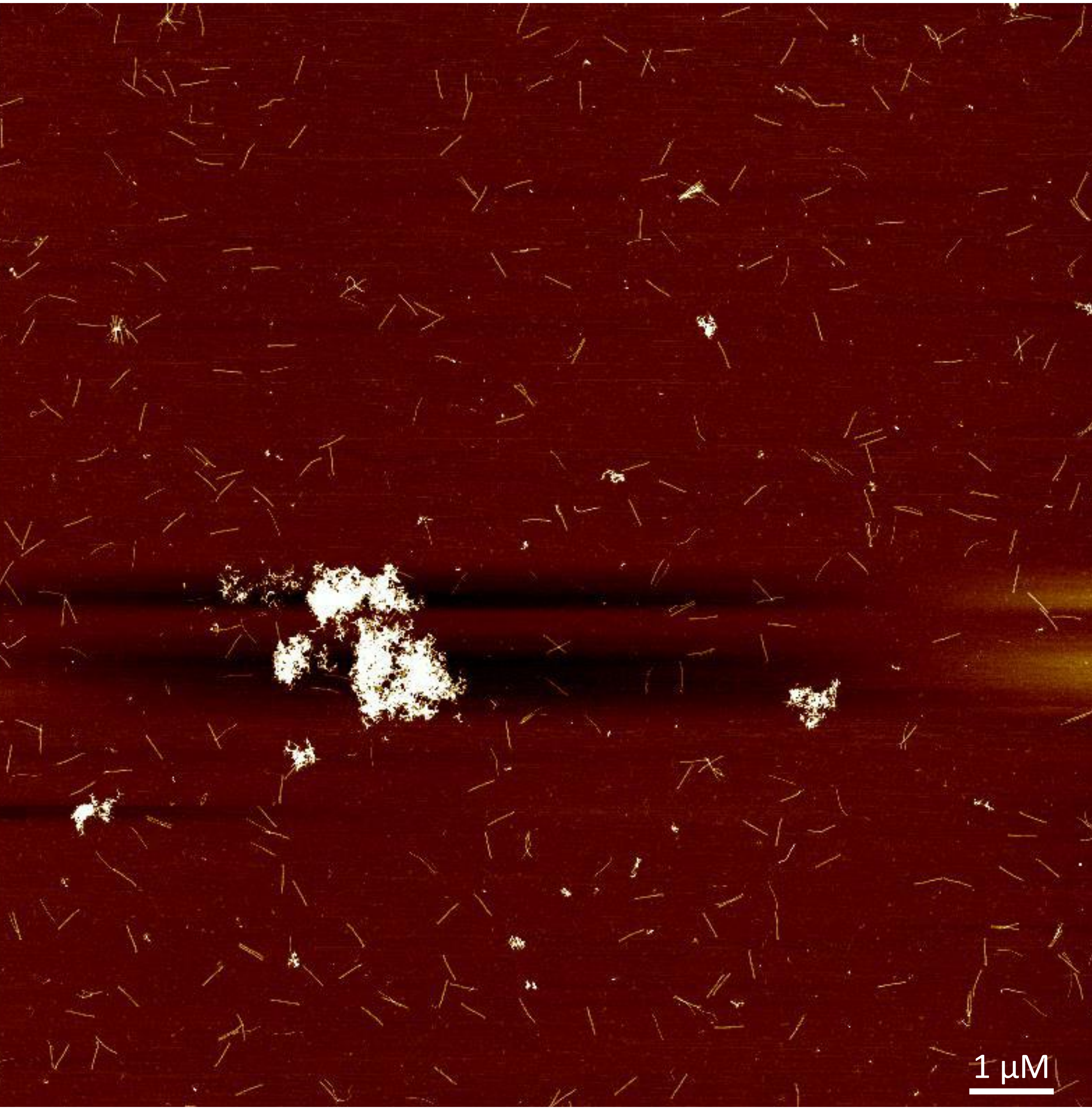

[SYBR Green I (SG)] = 0.4 μM
[Ethidium Bromide (EtBr)] = 0 μM

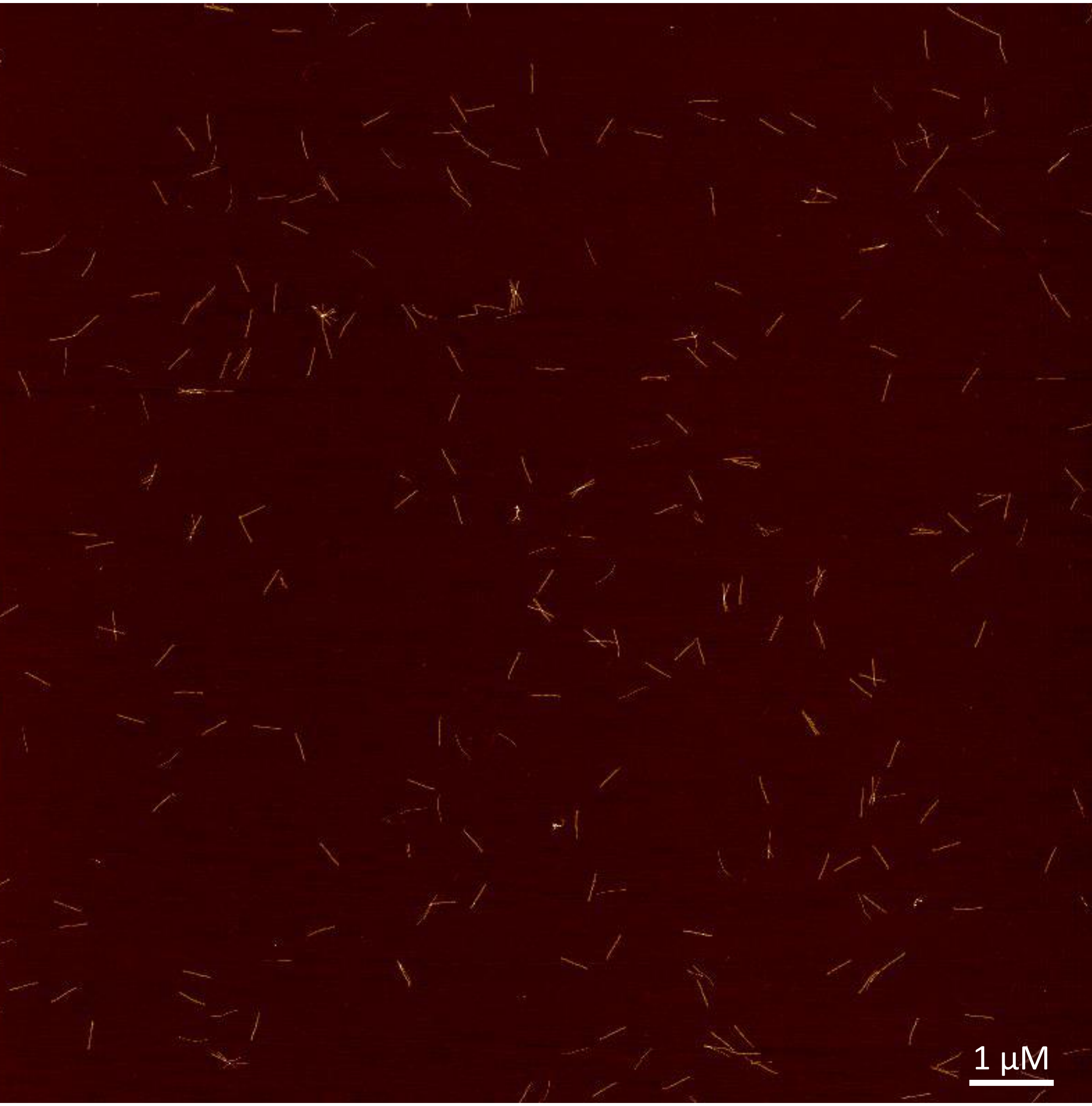

[SYBR Green I (SG)] = 0.4 µM
[Ethidium Bromide (EtBr)] = 0 µM

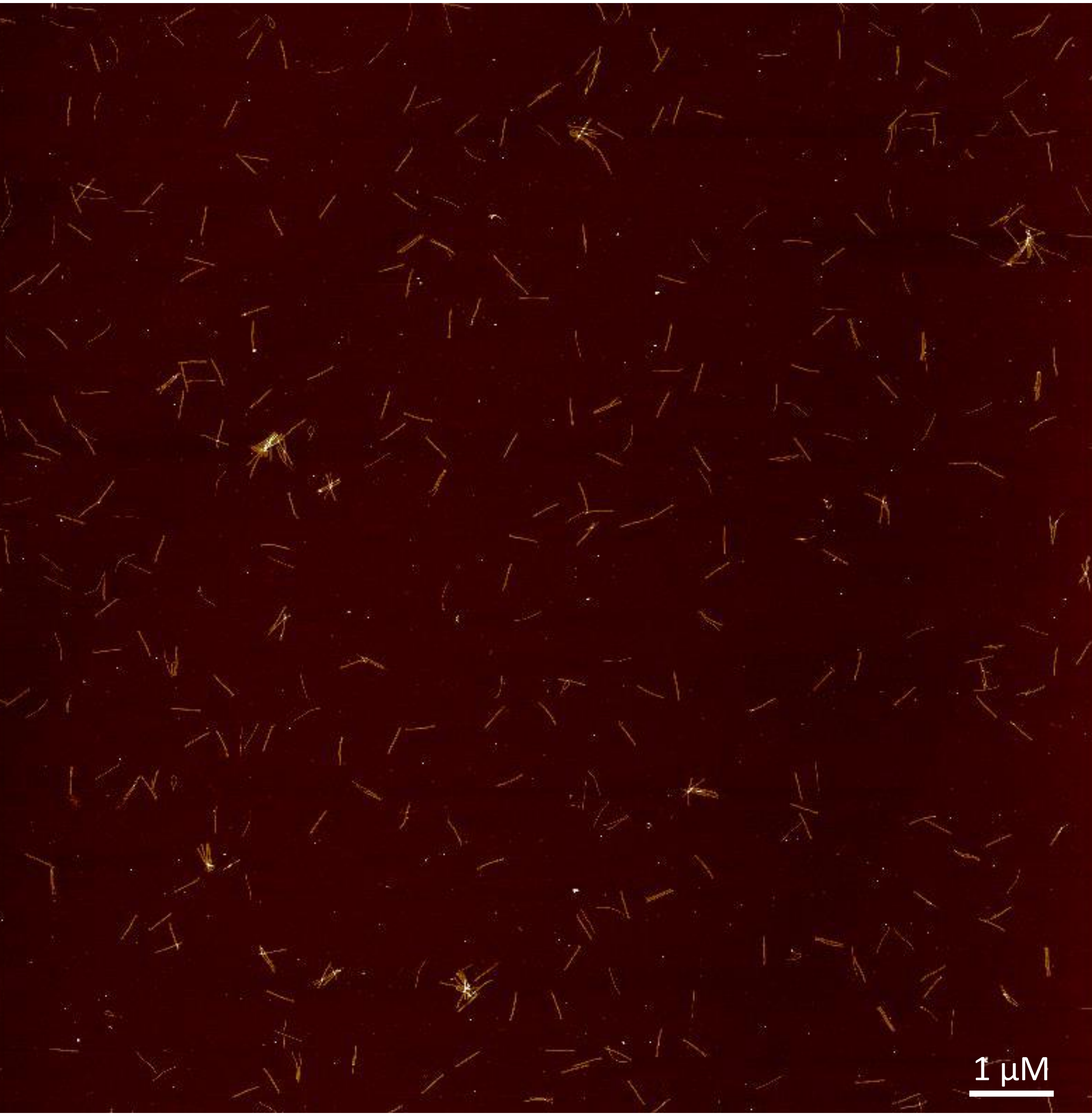

[SYBR Green I (SG)] = 1.2 µM
[Ethidium Bromide (EtBr)] = 0 µM

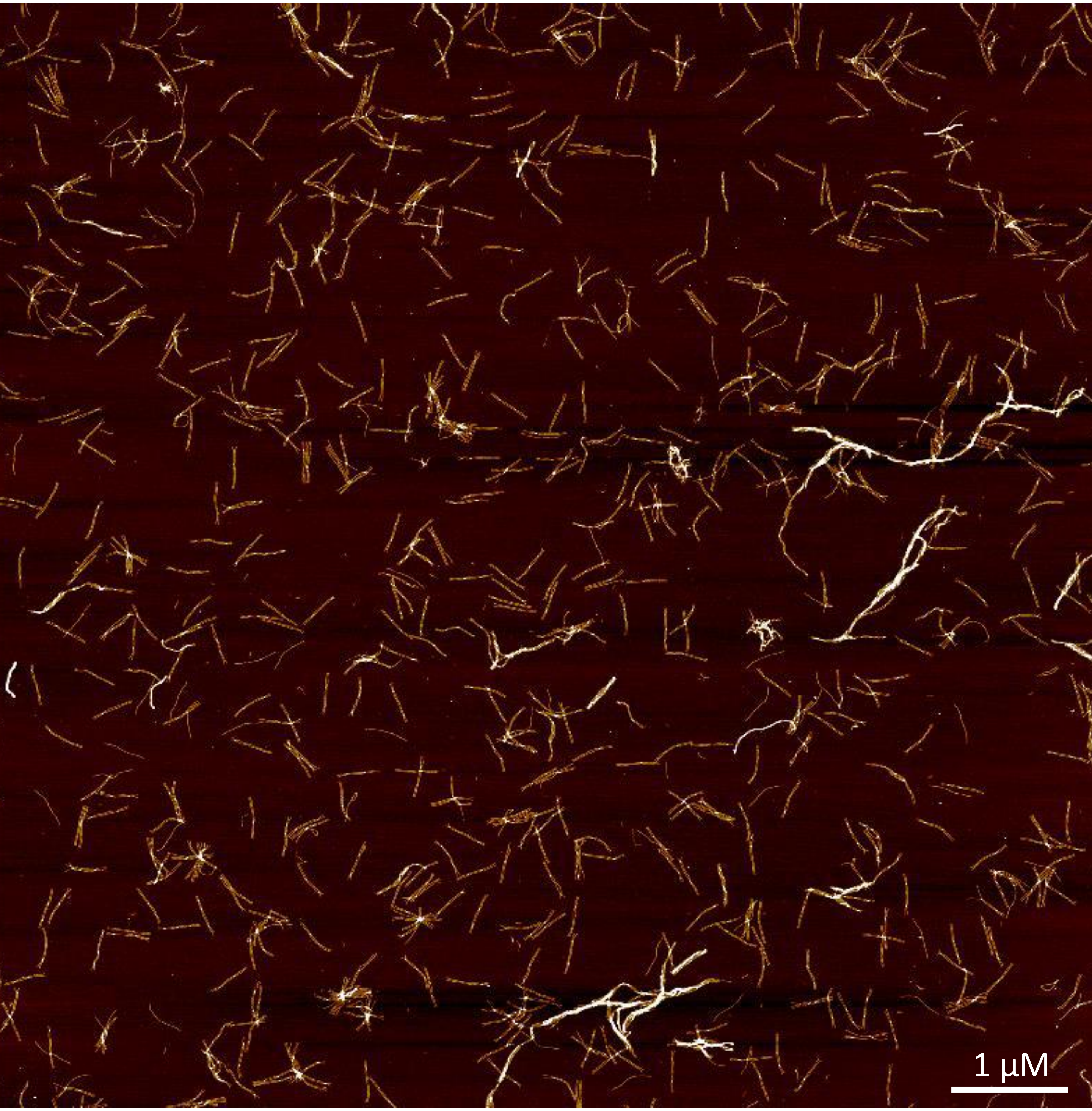

[SYBR Green I (SG)] = 2 µM
[Ethidium Bromide (EtBr)] = 0 µM

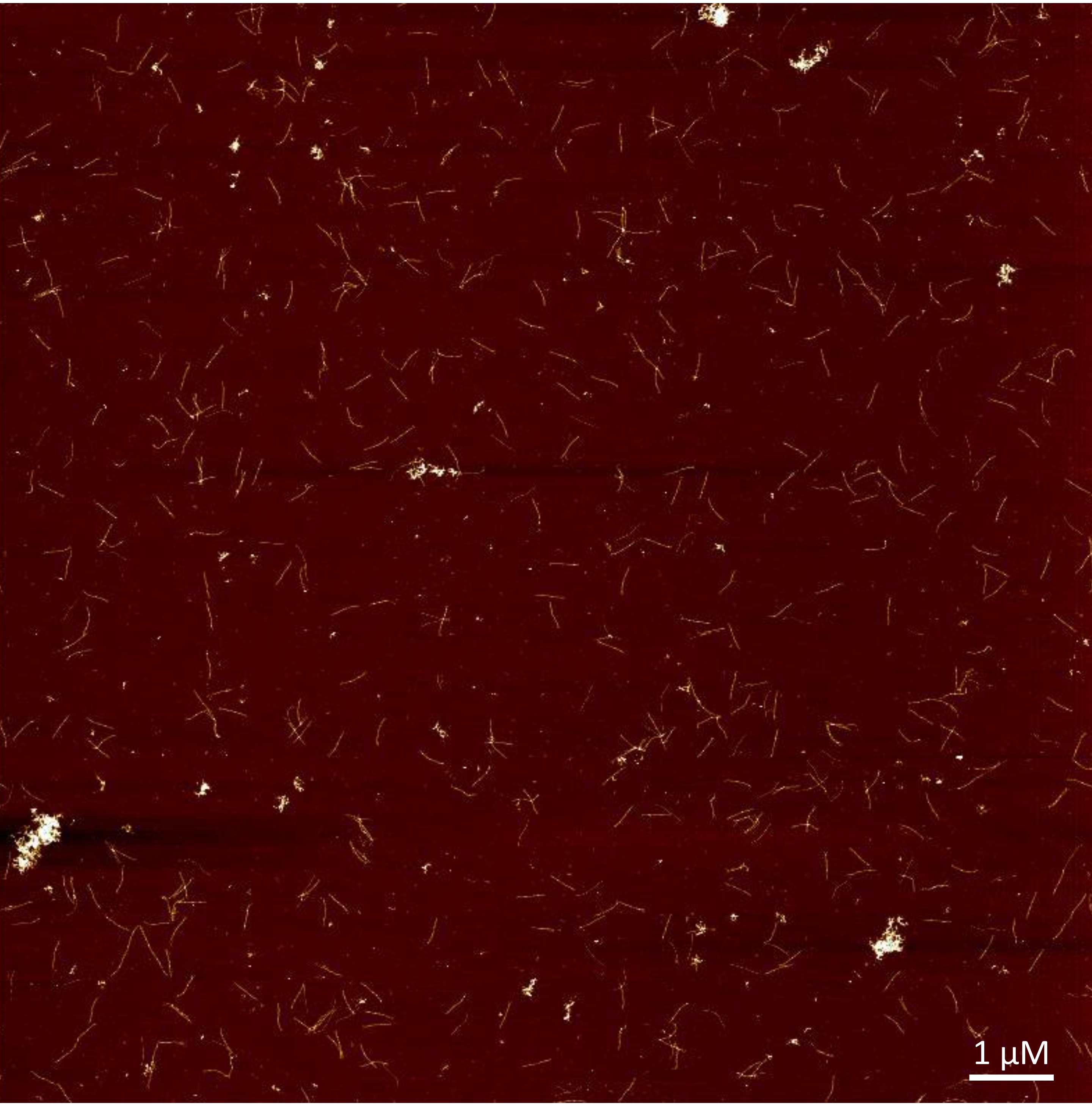

[SYBR Green I (SG)] = 3 µM
[Ethidium Bromide (EtBr)] = 0 µM

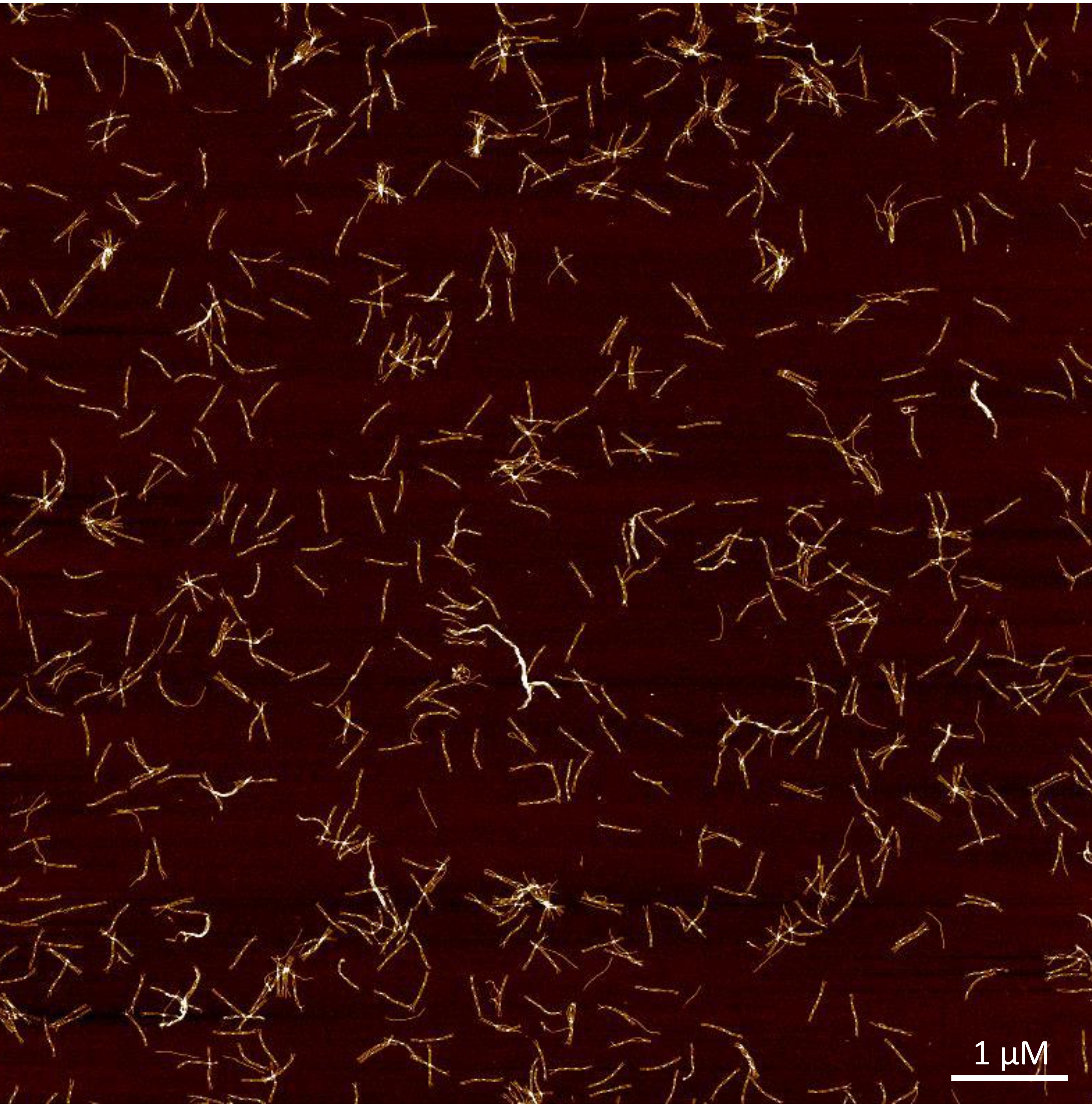

[SYBR Green I (SG)] = 4 μM
[Ethidium Bromide (EtBr)] = 0 μM

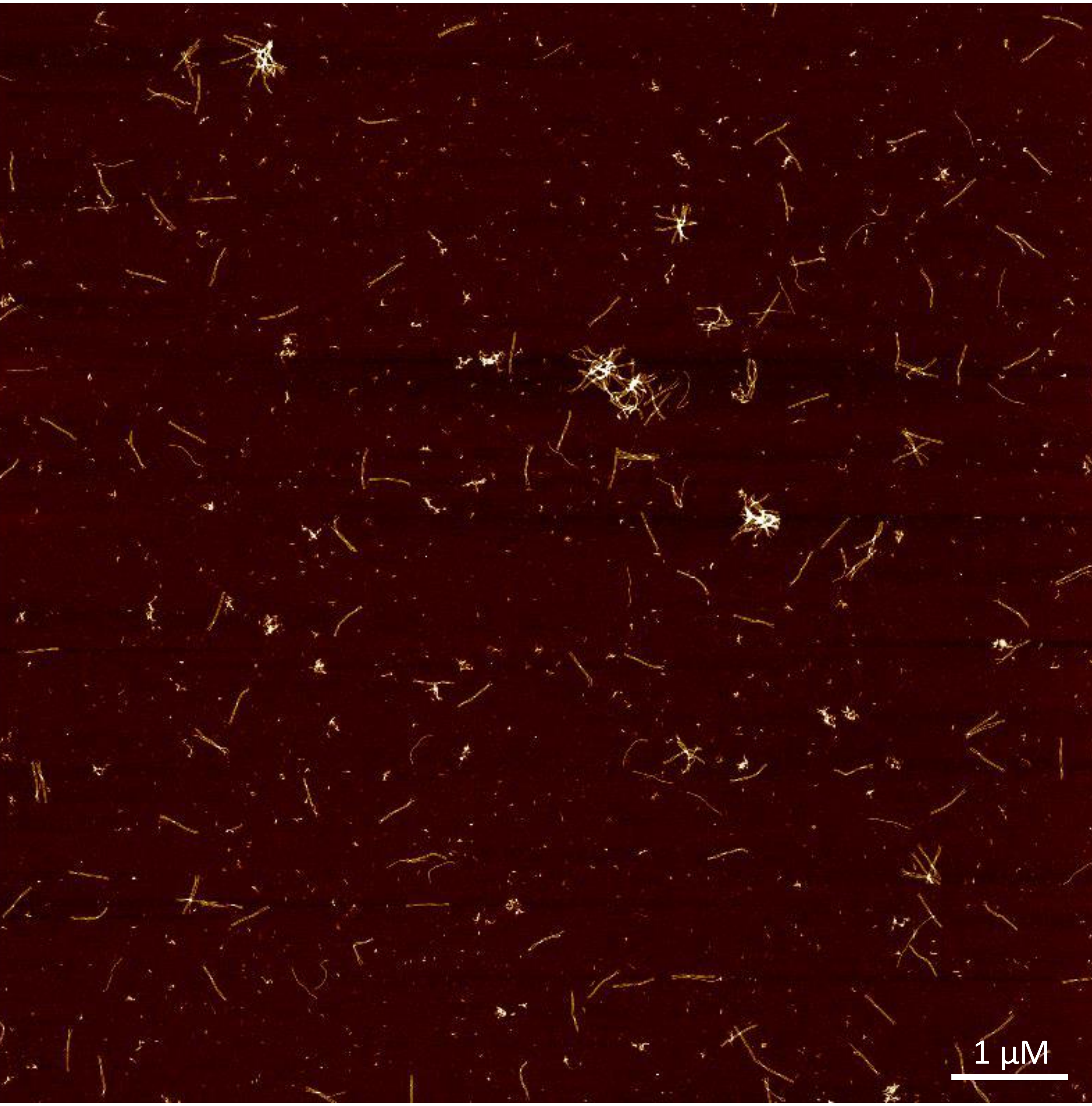

[SYBR Green I (SG)] = 4 µM
[Ethidium Bromide (EtBr)] = 0 µM

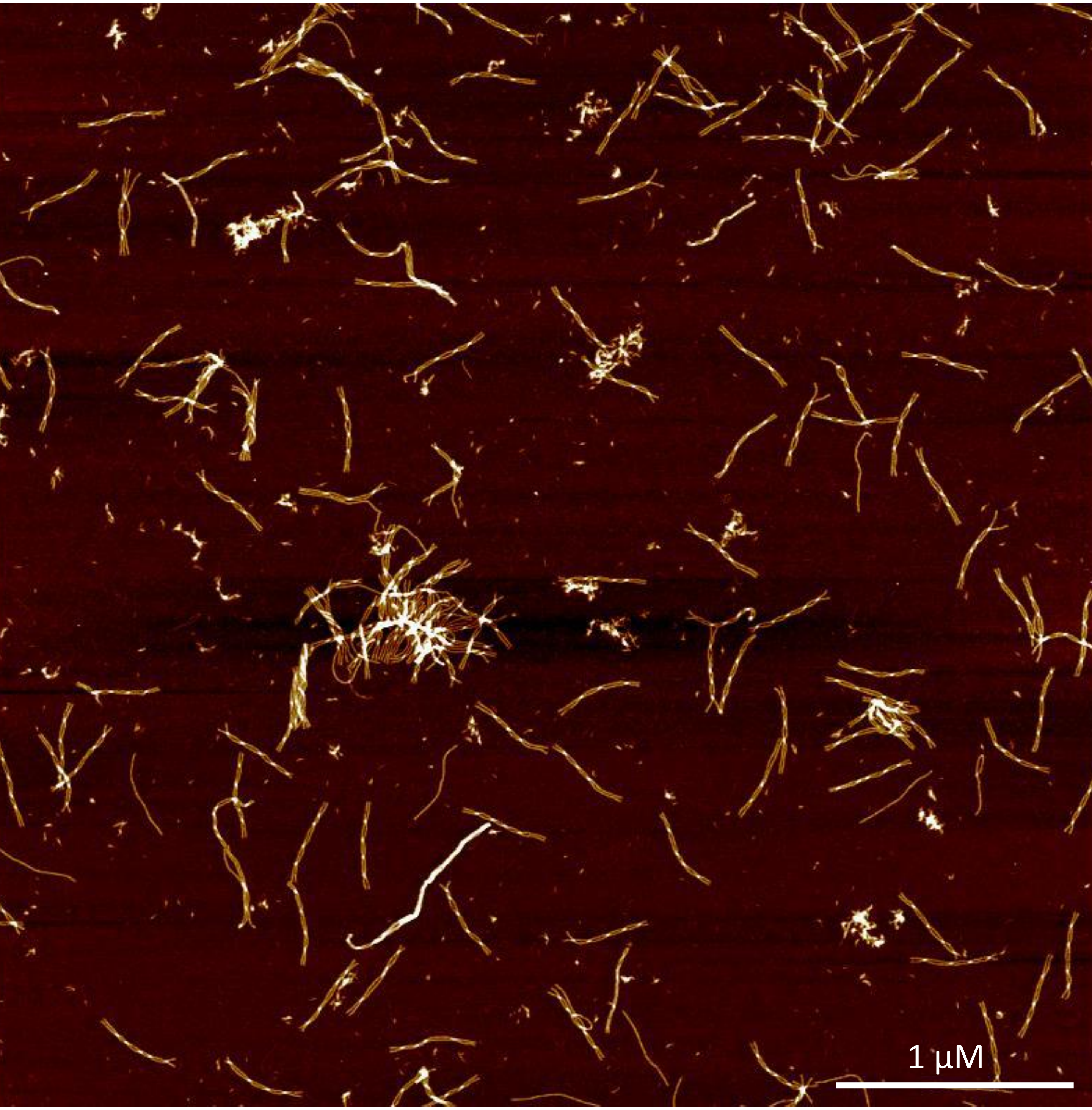

[SYBR Green I (SG)] = 4 μM
[Ethidium Bromide (EtBr)] = 0 μM

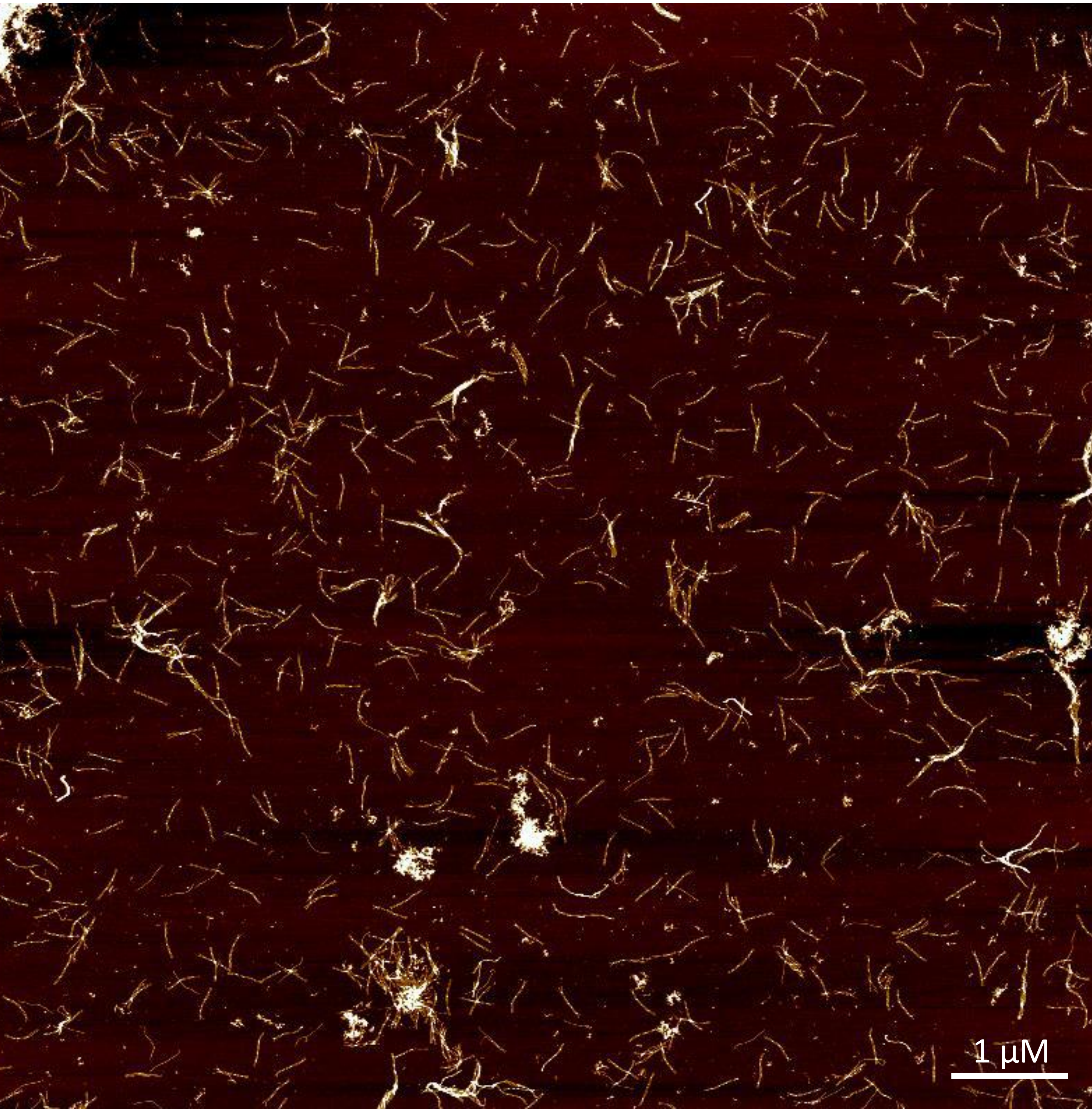

[SYBR Green I (SG)] = 4 μM
[Ethidium Bromide (EtBr)] = 0 μM

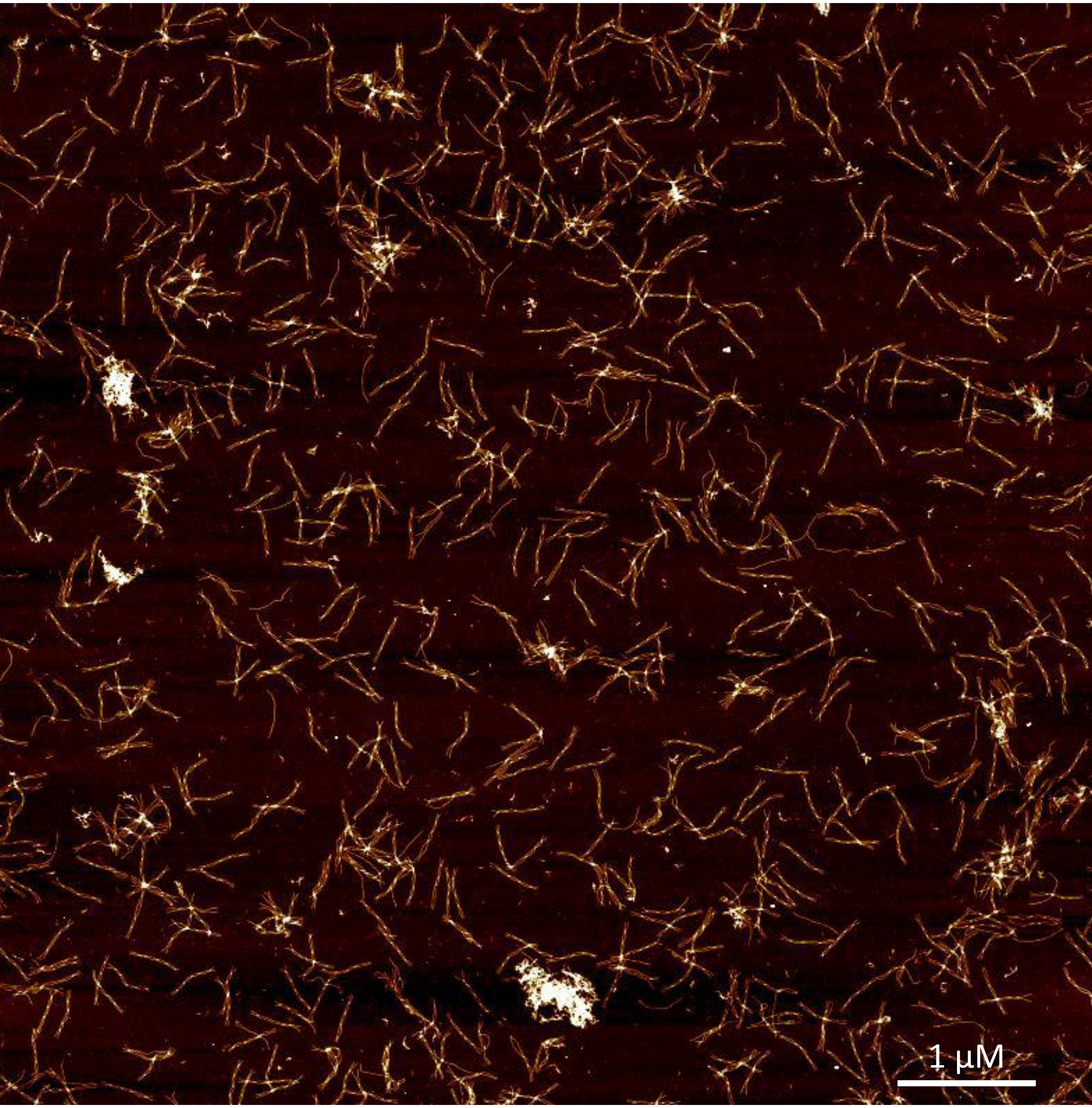

[SYBR Green I (SG)] = 4 µM
[Ethidium Bromide (EtBr)] = 0 µM

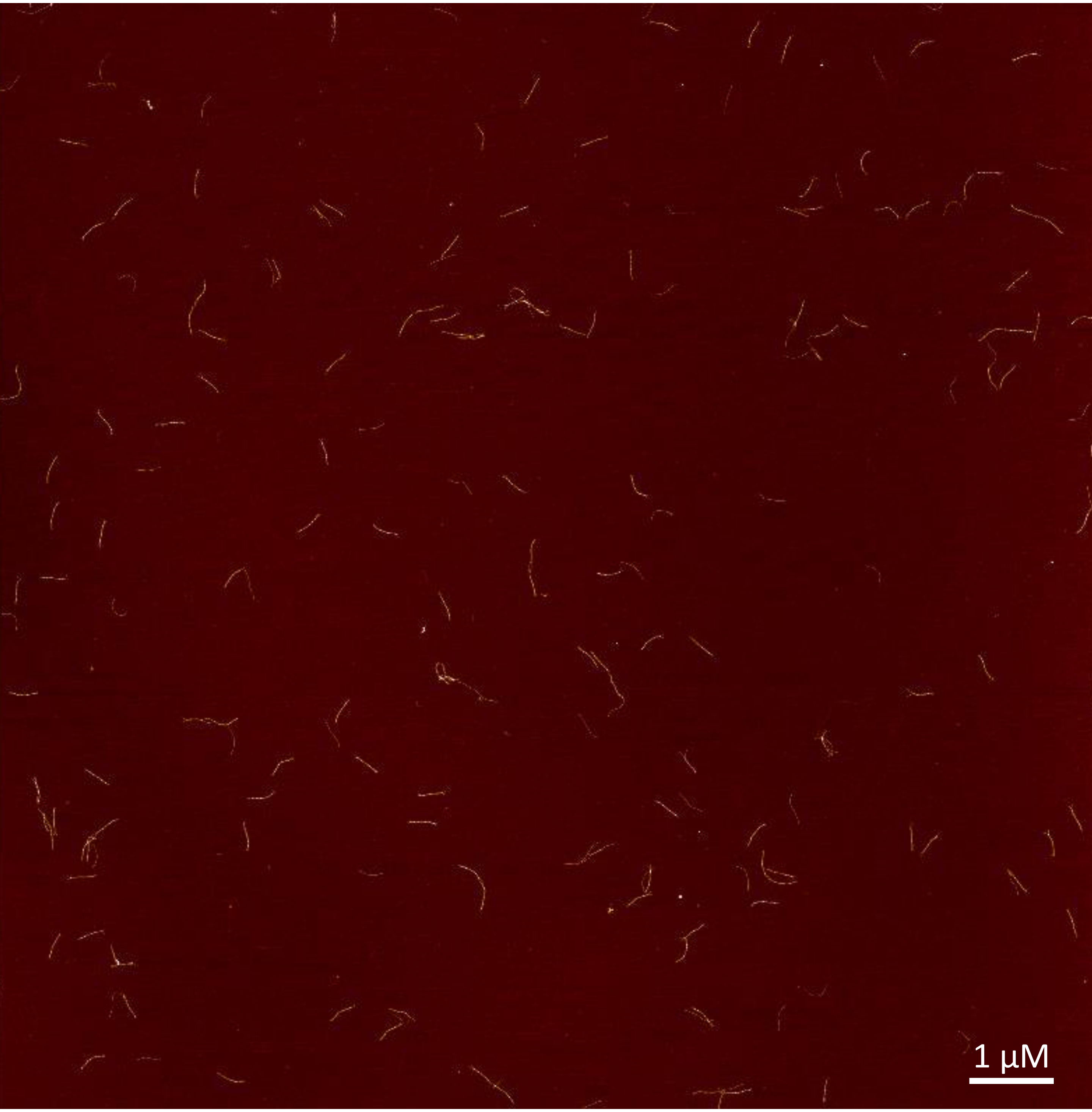

[SYBR Green I (SG)] = 4 μM
[Ethidium Bromide (EtBr)] = 0 μM

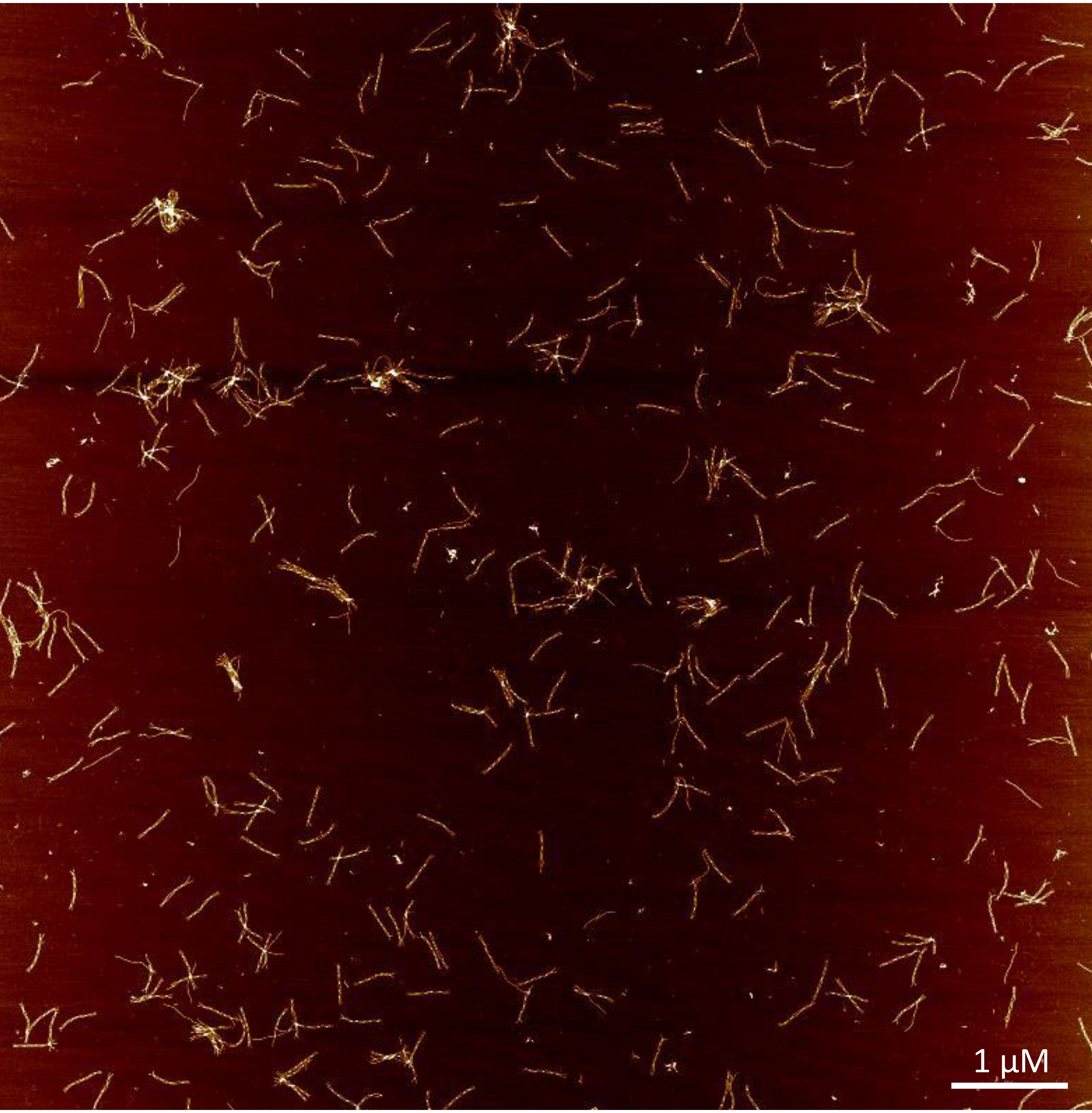

[SYBR Green I (SG)] = 4 μM
[Ethidium Bromide (EtBr)] = 0 μM

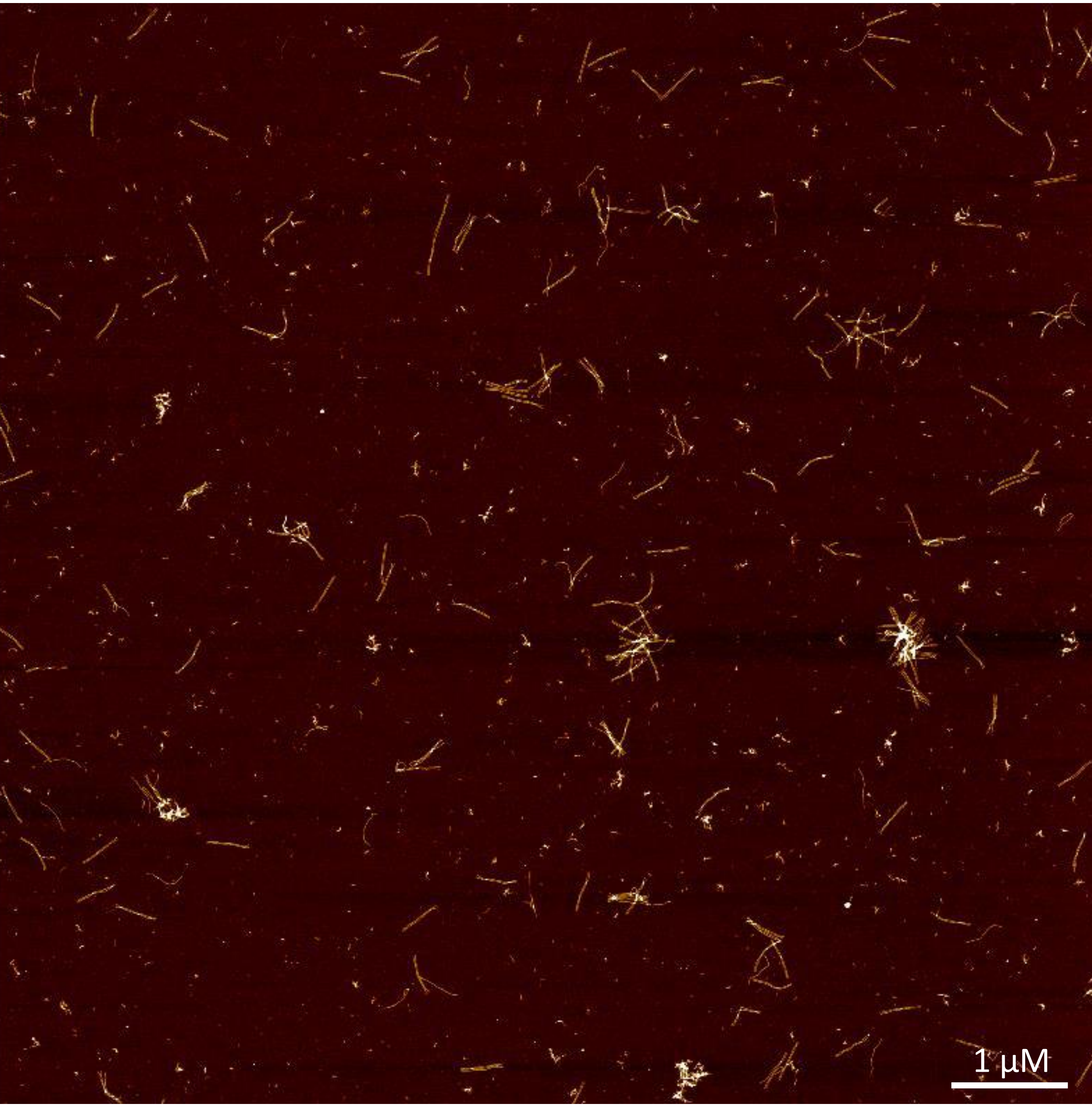

[SYBR Green I (SG)] = 4 μM
[Ethidium Bromide (EtBr)] = 0 μM

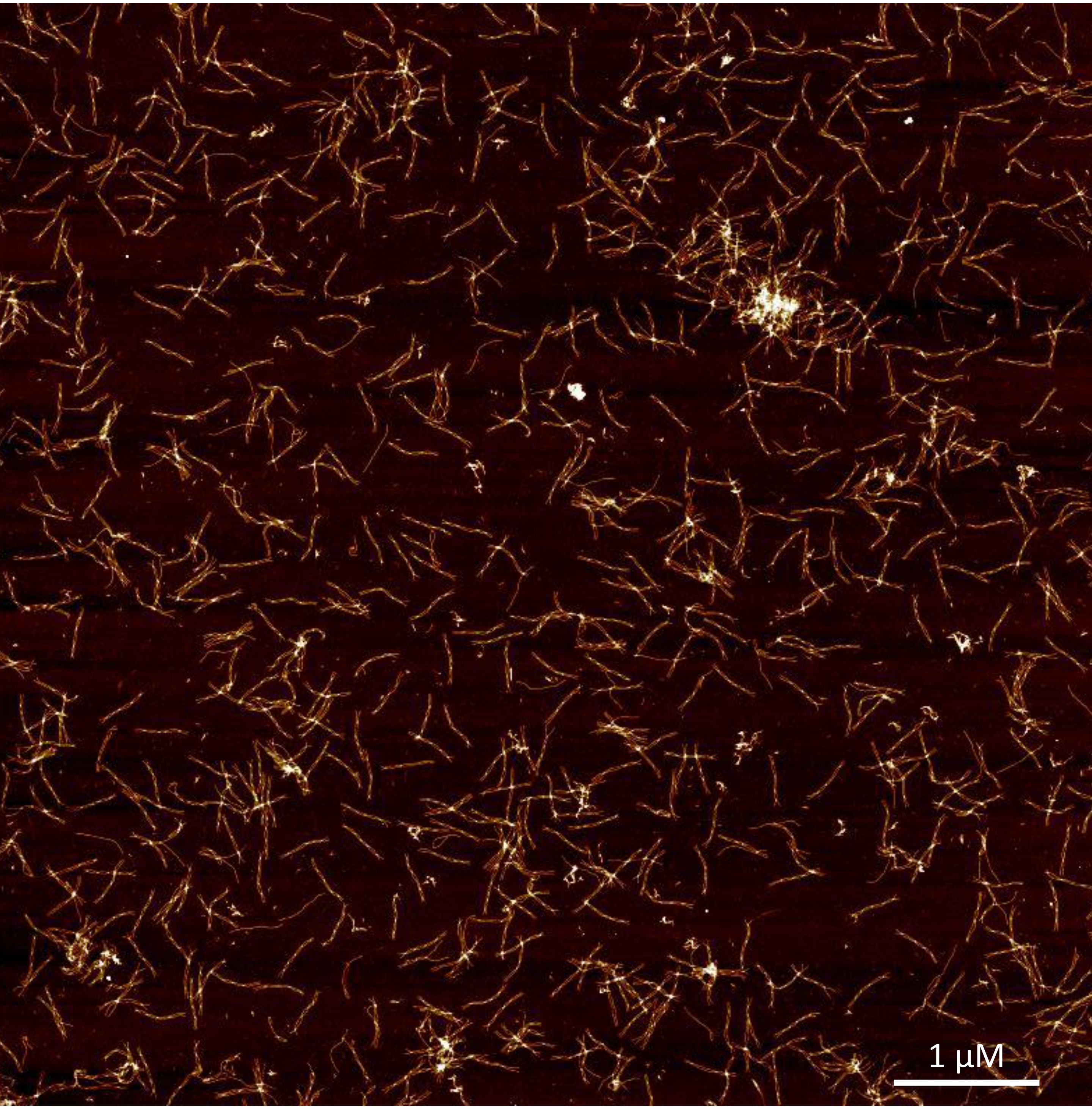

[SYBR Green I (SG)] = 8 µM
[Ethidium Bromide (EtBr)] = 0 µM

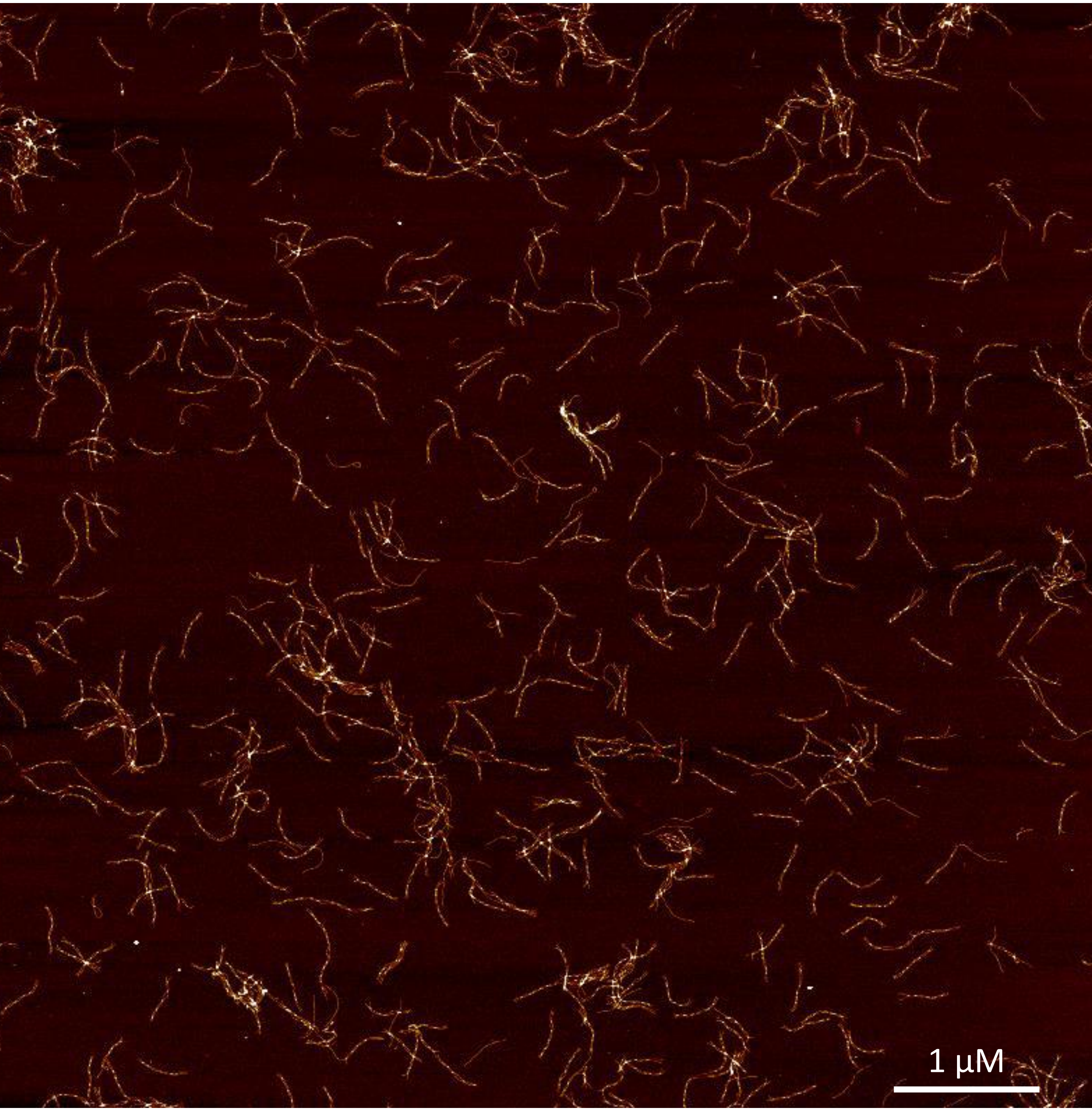

[SYBR Green I (SG)] = 16 μM
[Ethidium Bromide (EtBr)] = 0 μM

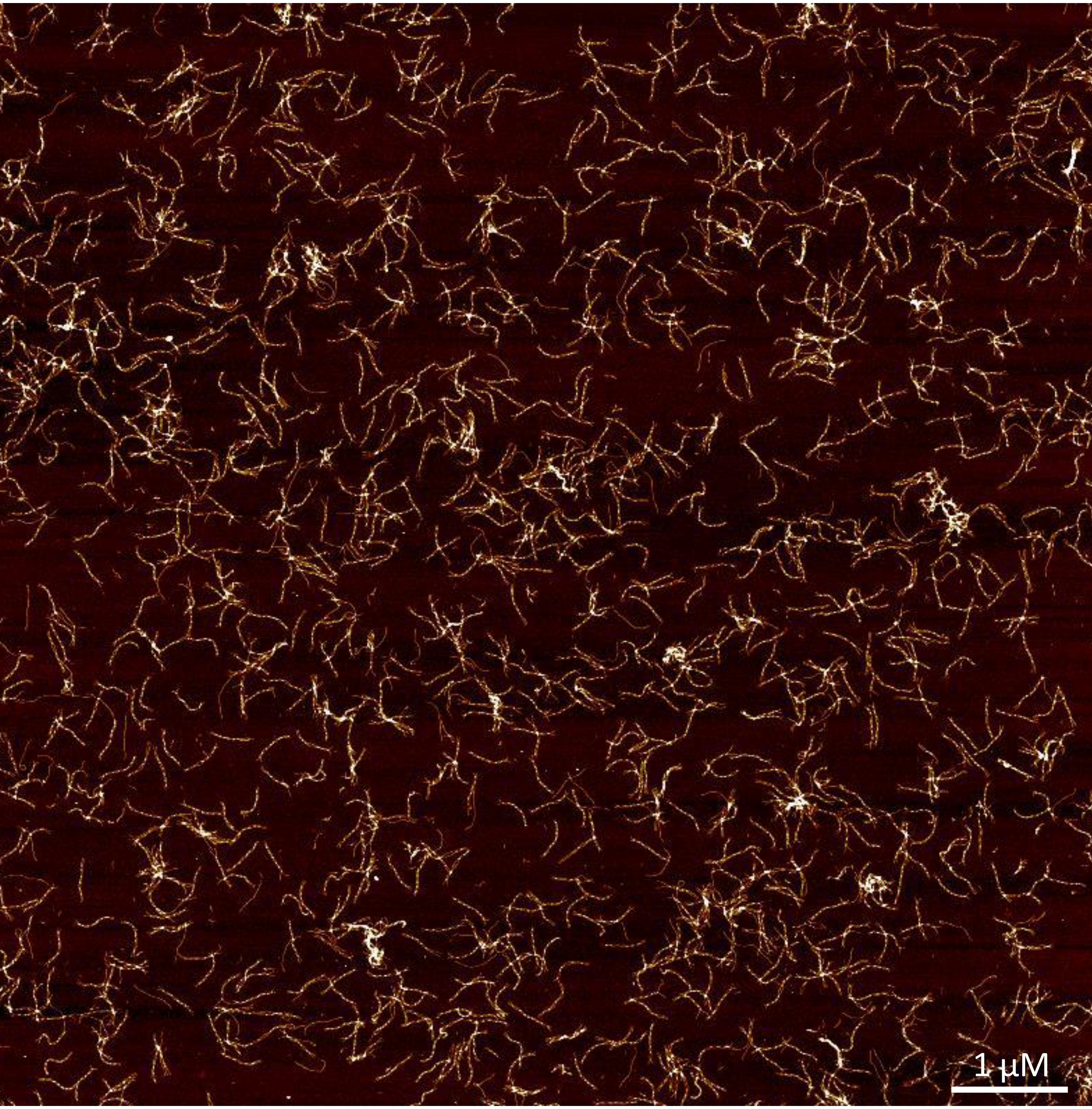

[SYBR Green I (SG)] = 20 μM
[Ethidium Bromide (EtBr)] = 0 μM

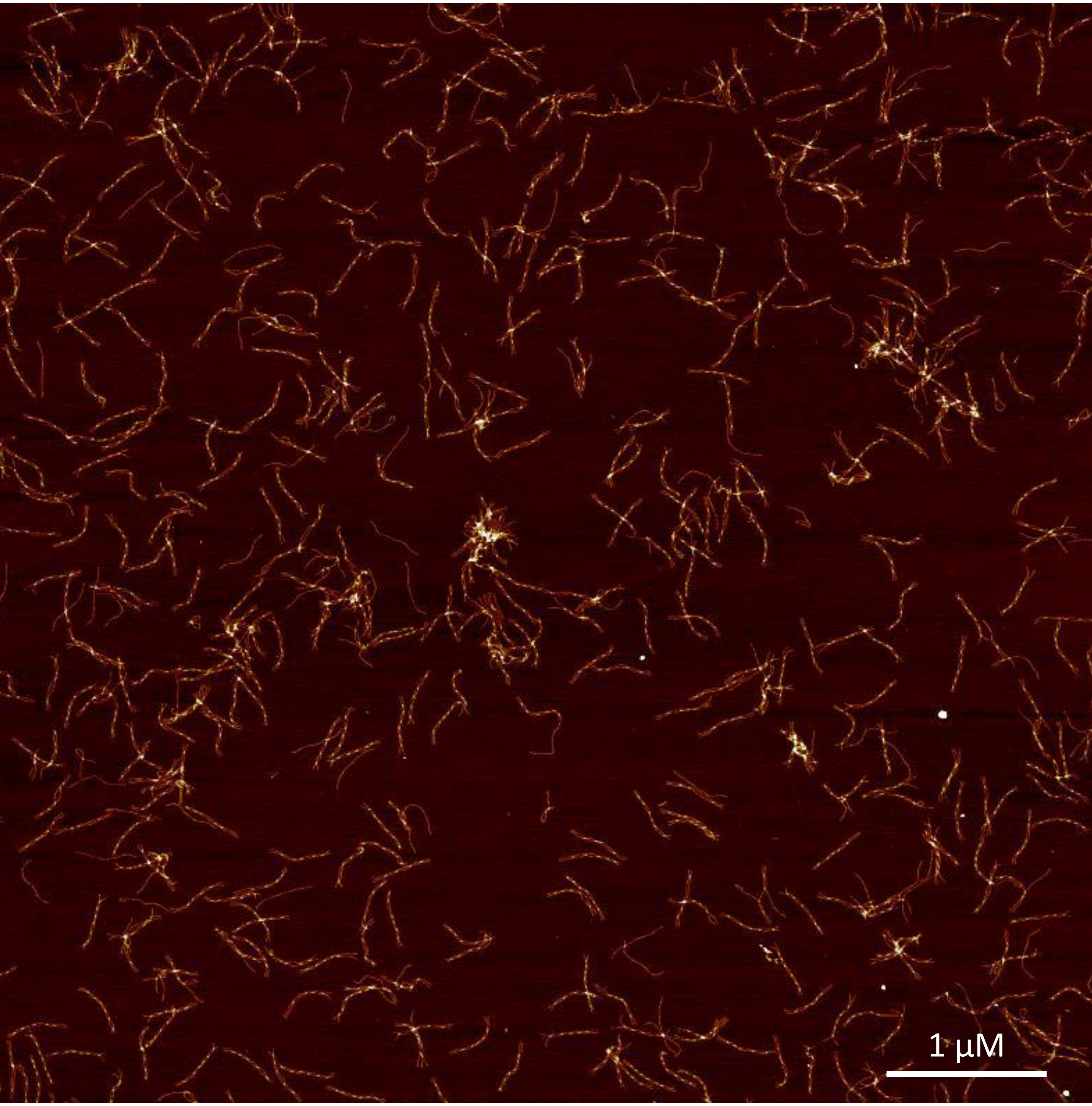

[SYBR Green I (SG)] = 20 µM
[Ethidium Bromide (EtBr)] = 0 µM

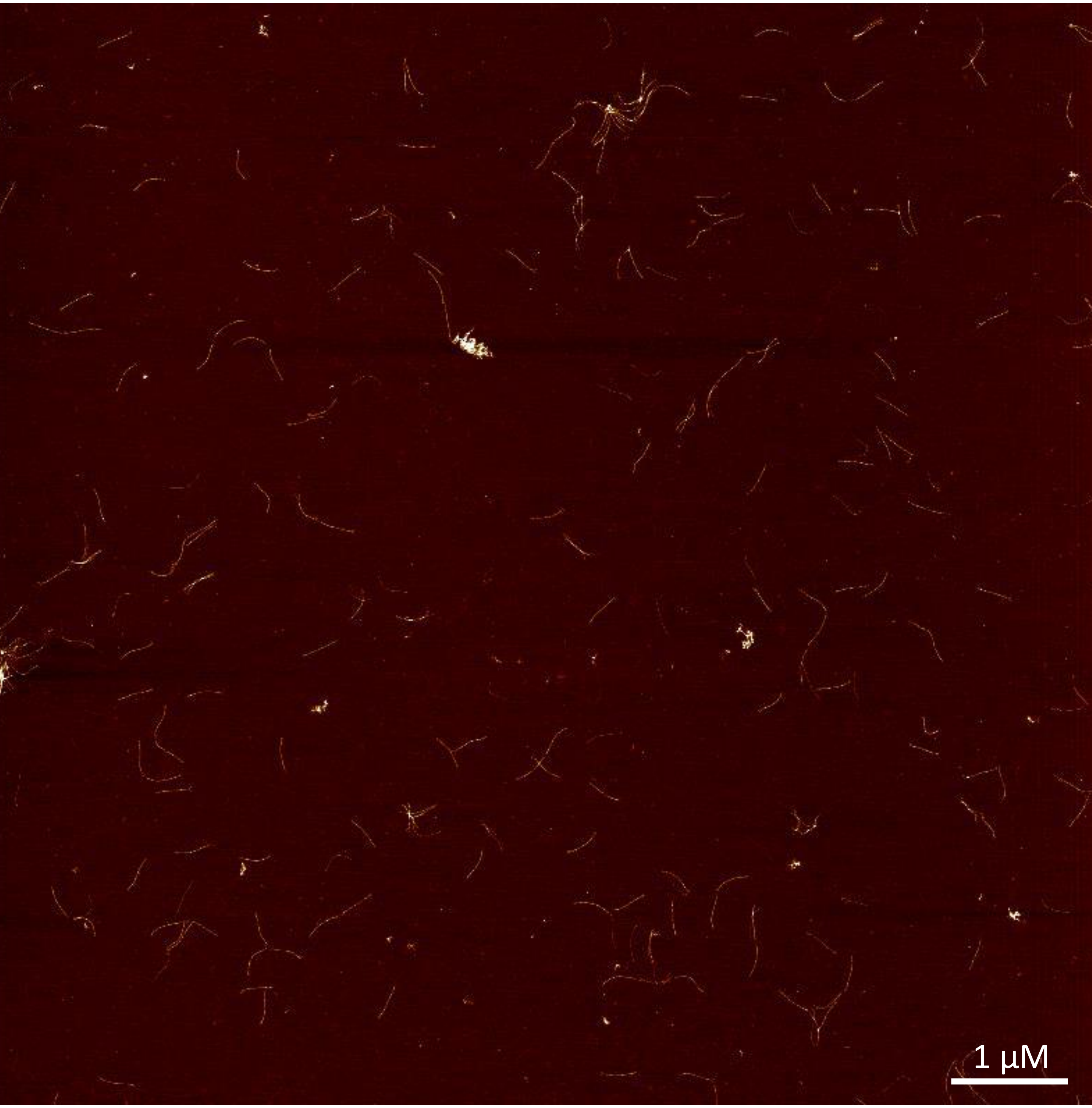

[SYBR Green I (SG)] = 20 μM
[Ethidium Bromide (EtBr)] = 0 μM

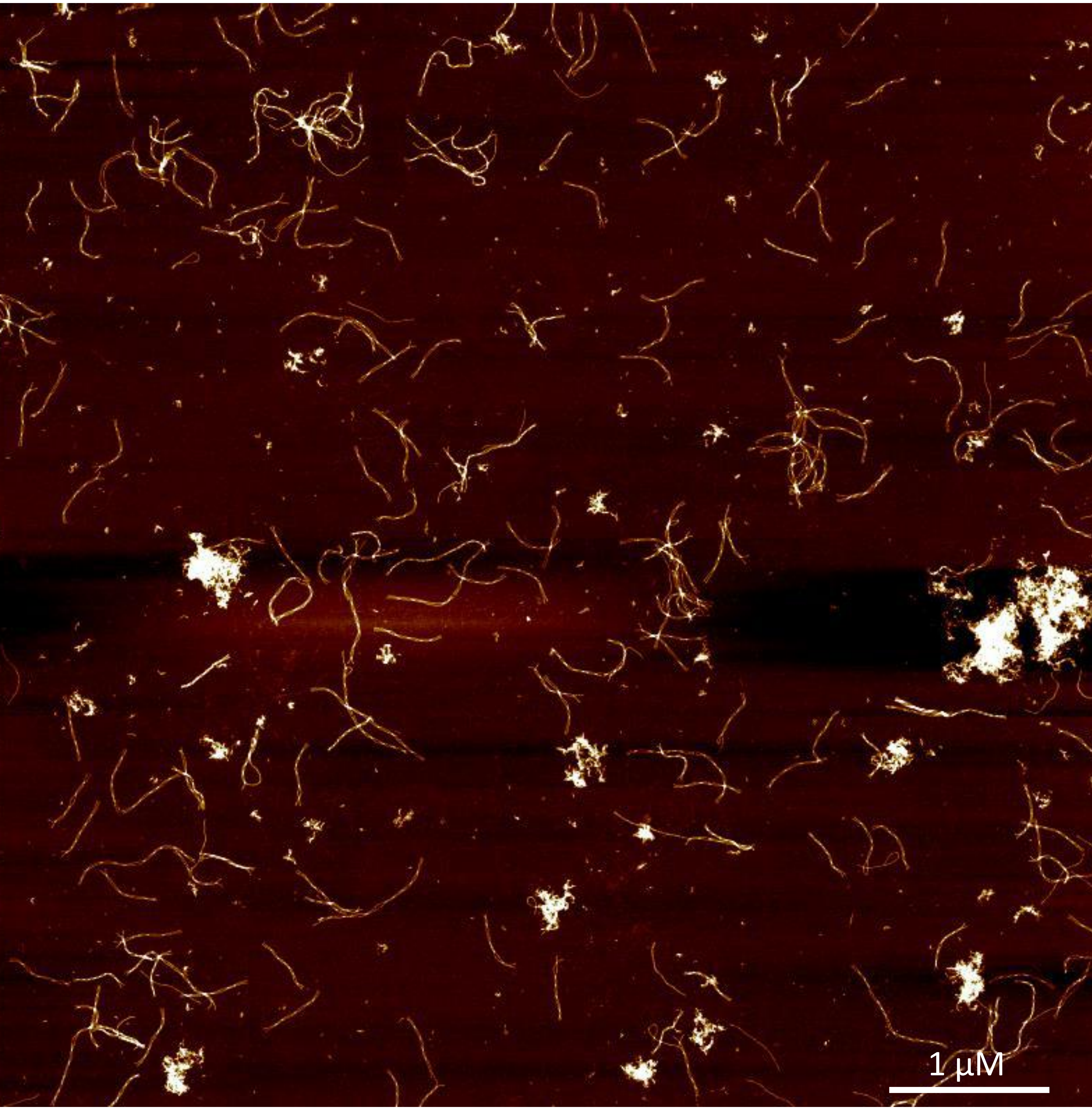

[SYBR Green I (SG)] = 20 µM
[Ethidium Bromide (EtBr)] = 0 µM

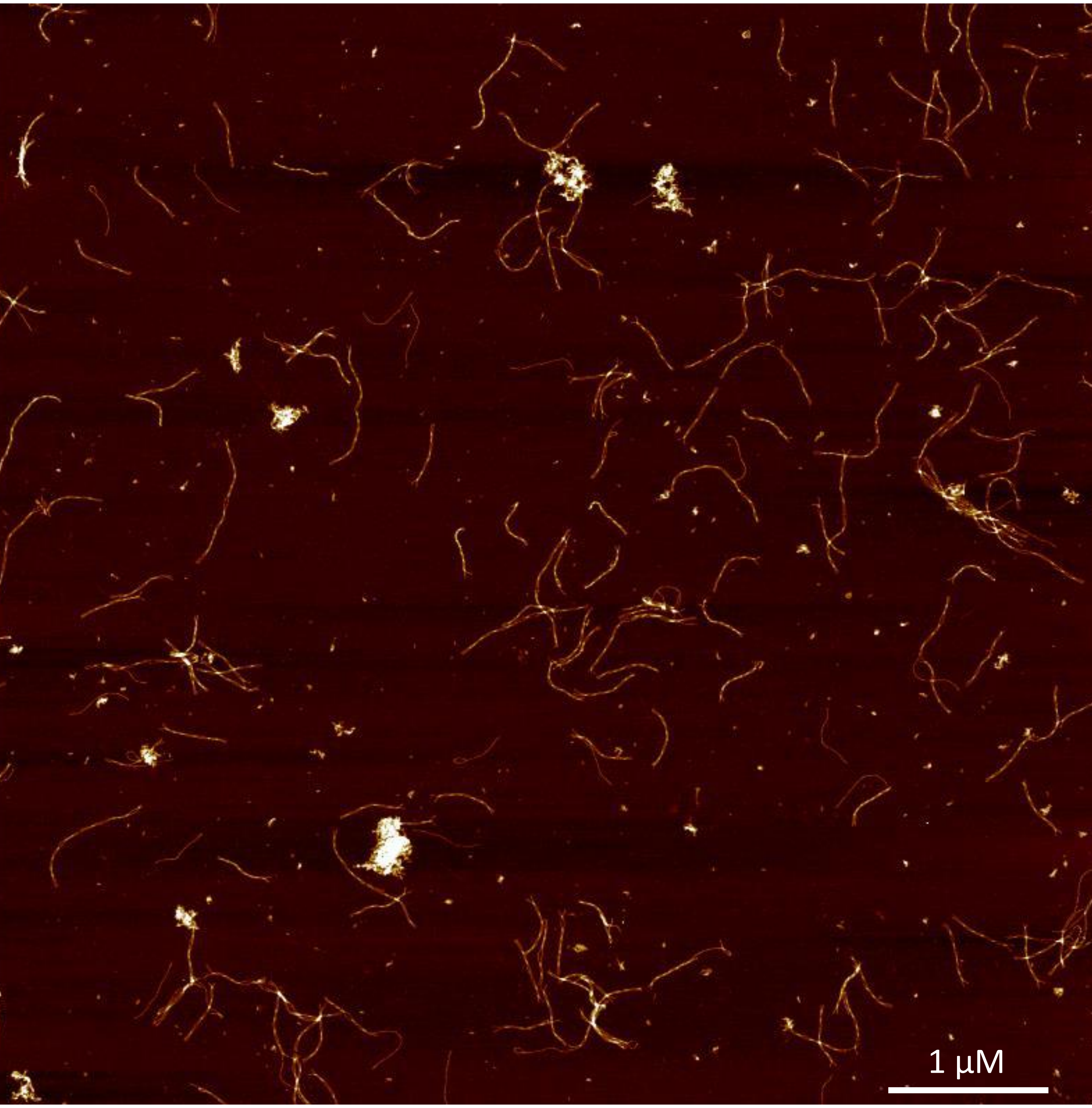

[SYBR Green I (SG)] = 20 µM
[Ethidium Bromide (EtBr)] = 0 µM

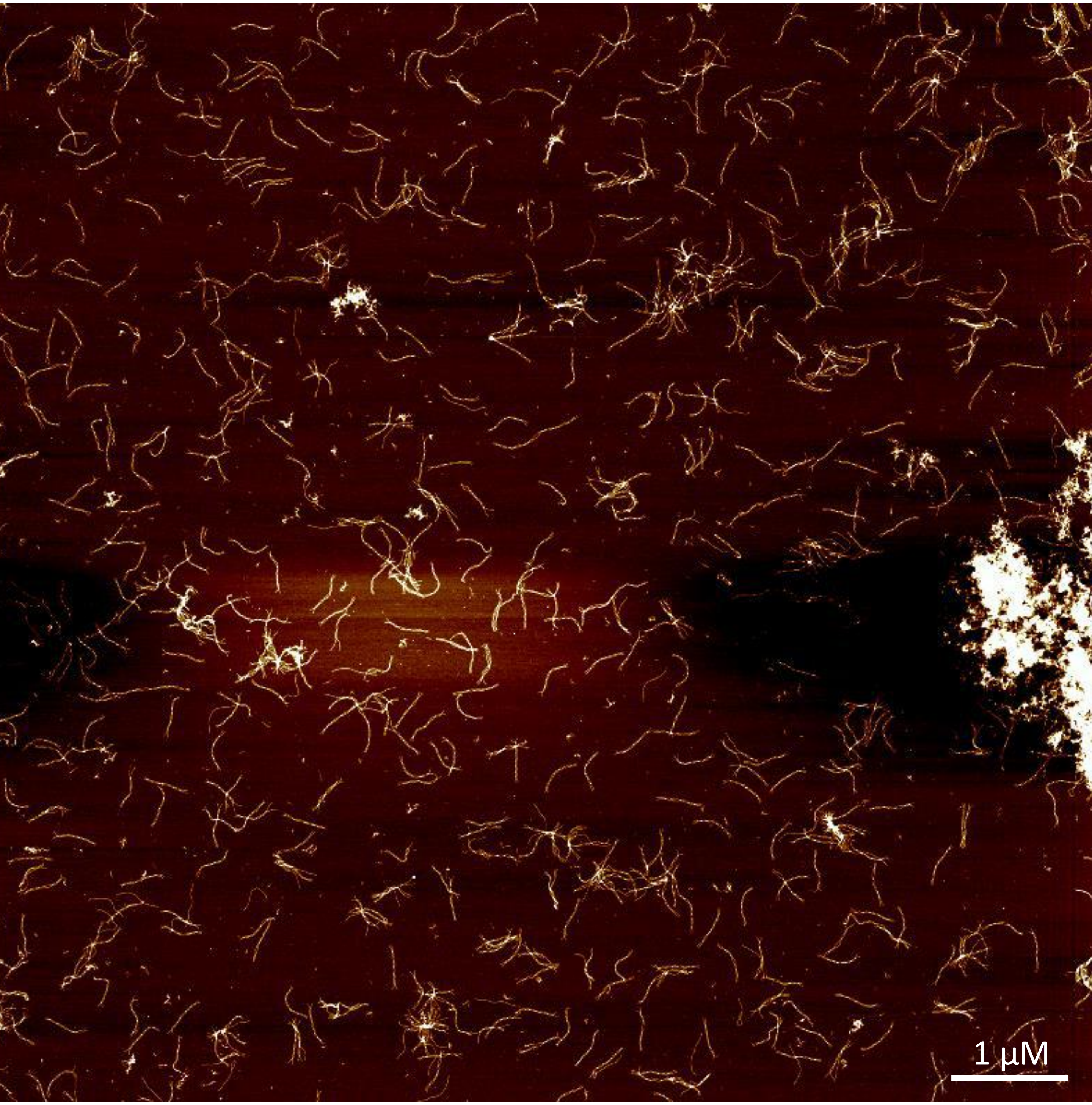

[SYBR Green I (SG)] = 20 μM
[Ethidium Bromide (EtBr)] = 0 μM

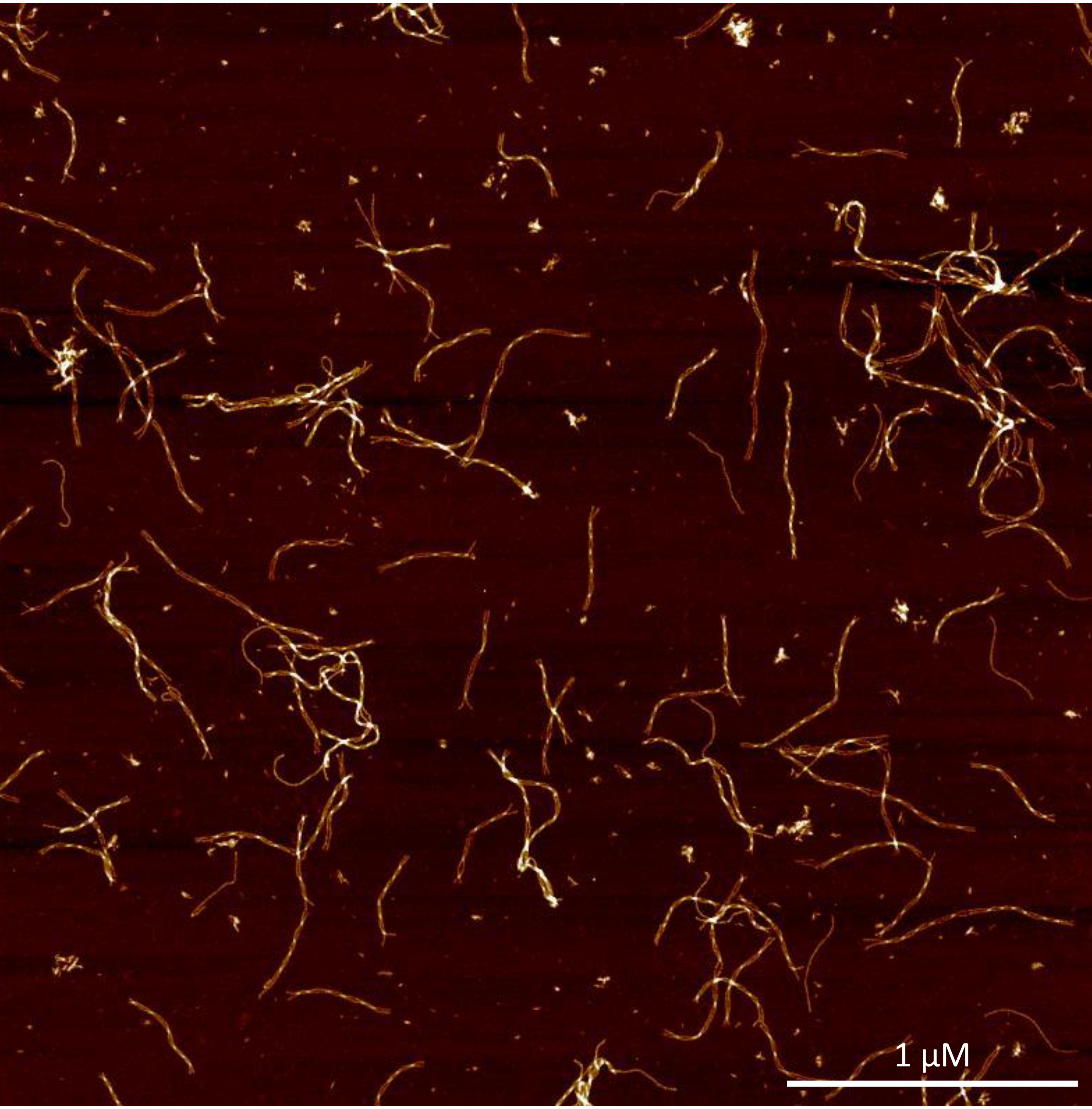

Dialyzed sample

Initial [SYBR Green I (SG)] = 20 µM
[Ethidium Bromide (EtBr)] = 0 µM

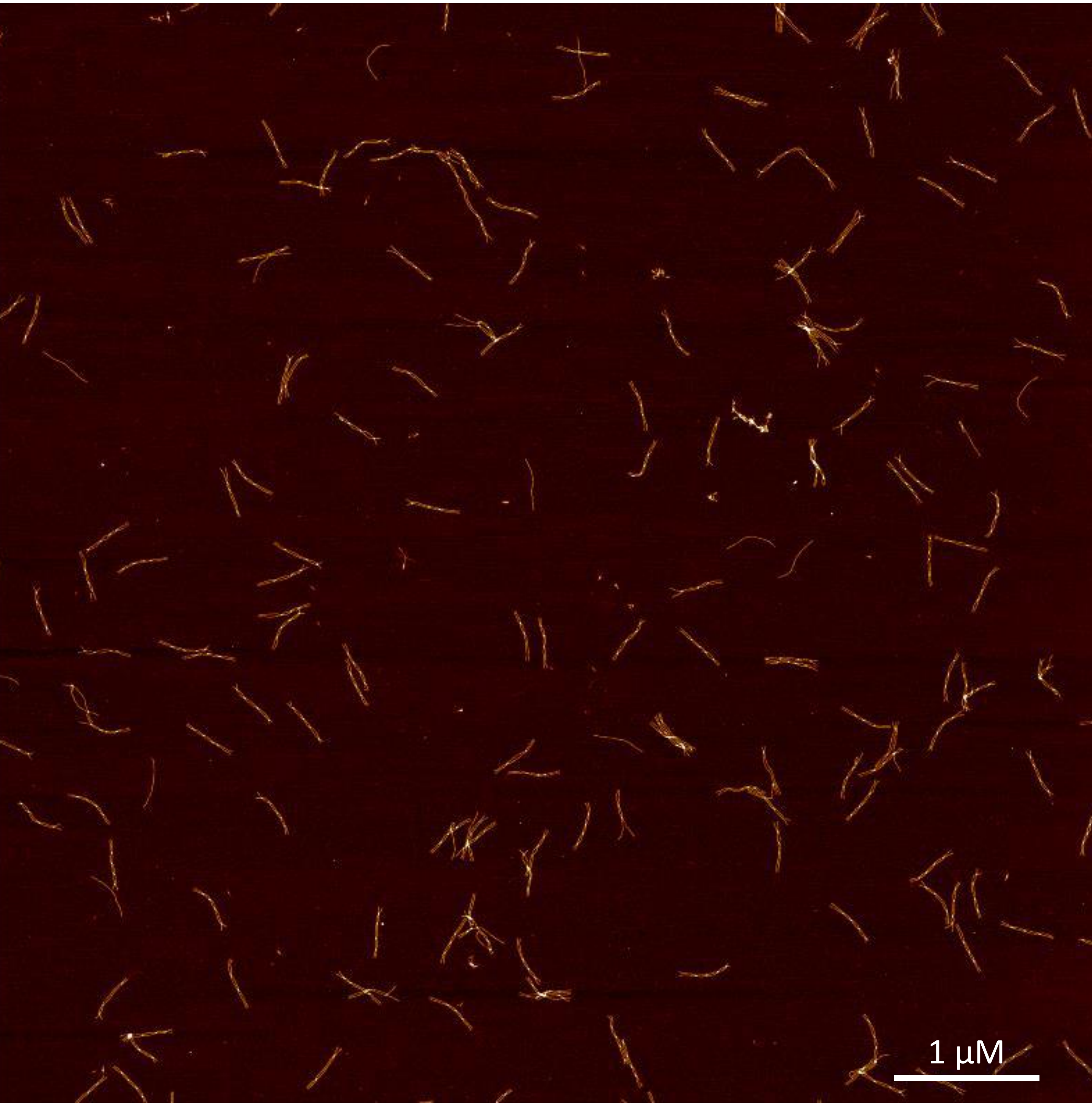

Dialyzed sample

Initial [SYBR Green I (SG)] = 20 µM
[Ethidium Bromide (EtBr)] = 0 µM

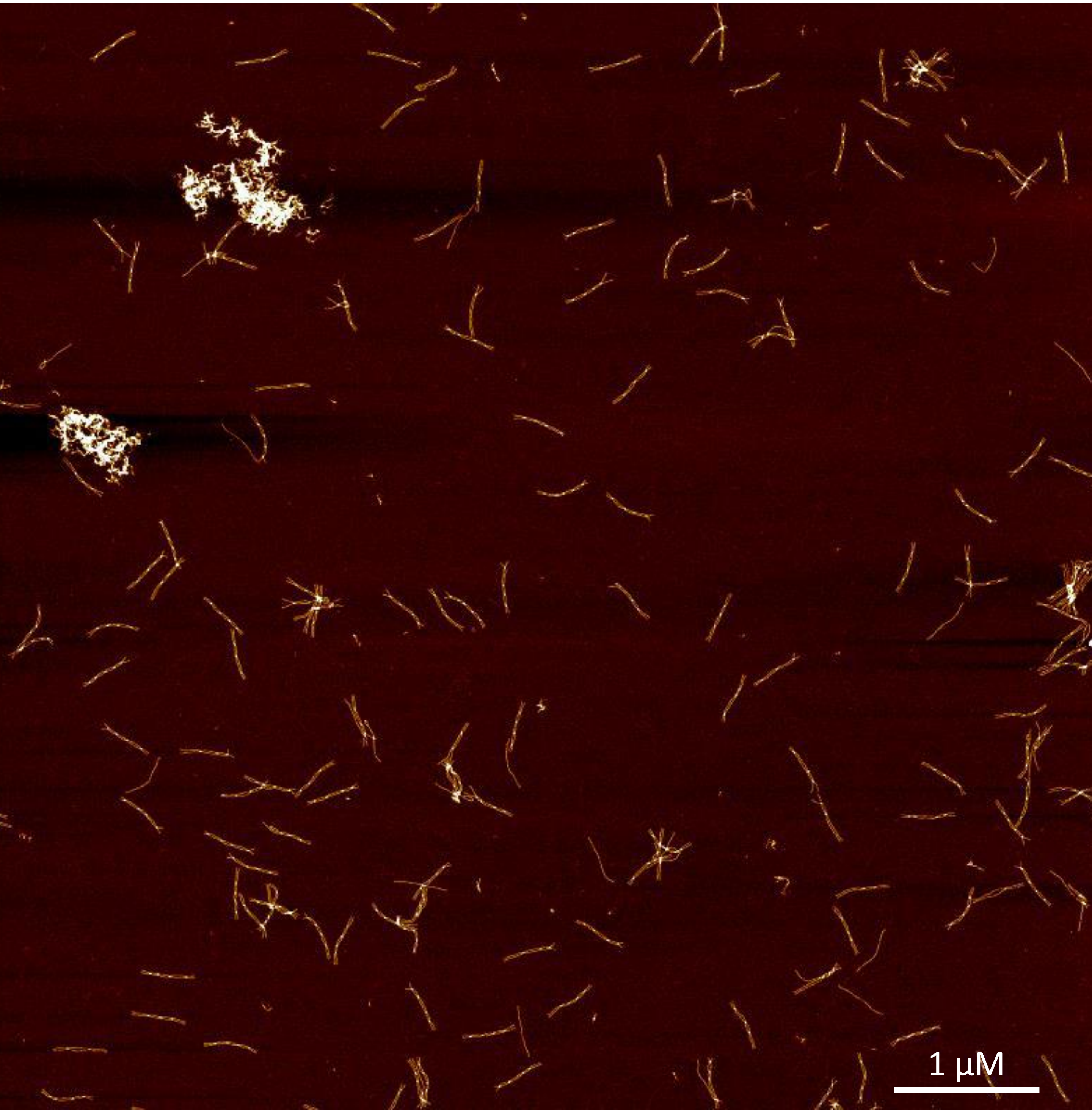

Dialyzed sample

Initial [SYBR Green I (SG)] = 20 µM
[Ethidium Bromide (EtBr)] = 0 µM

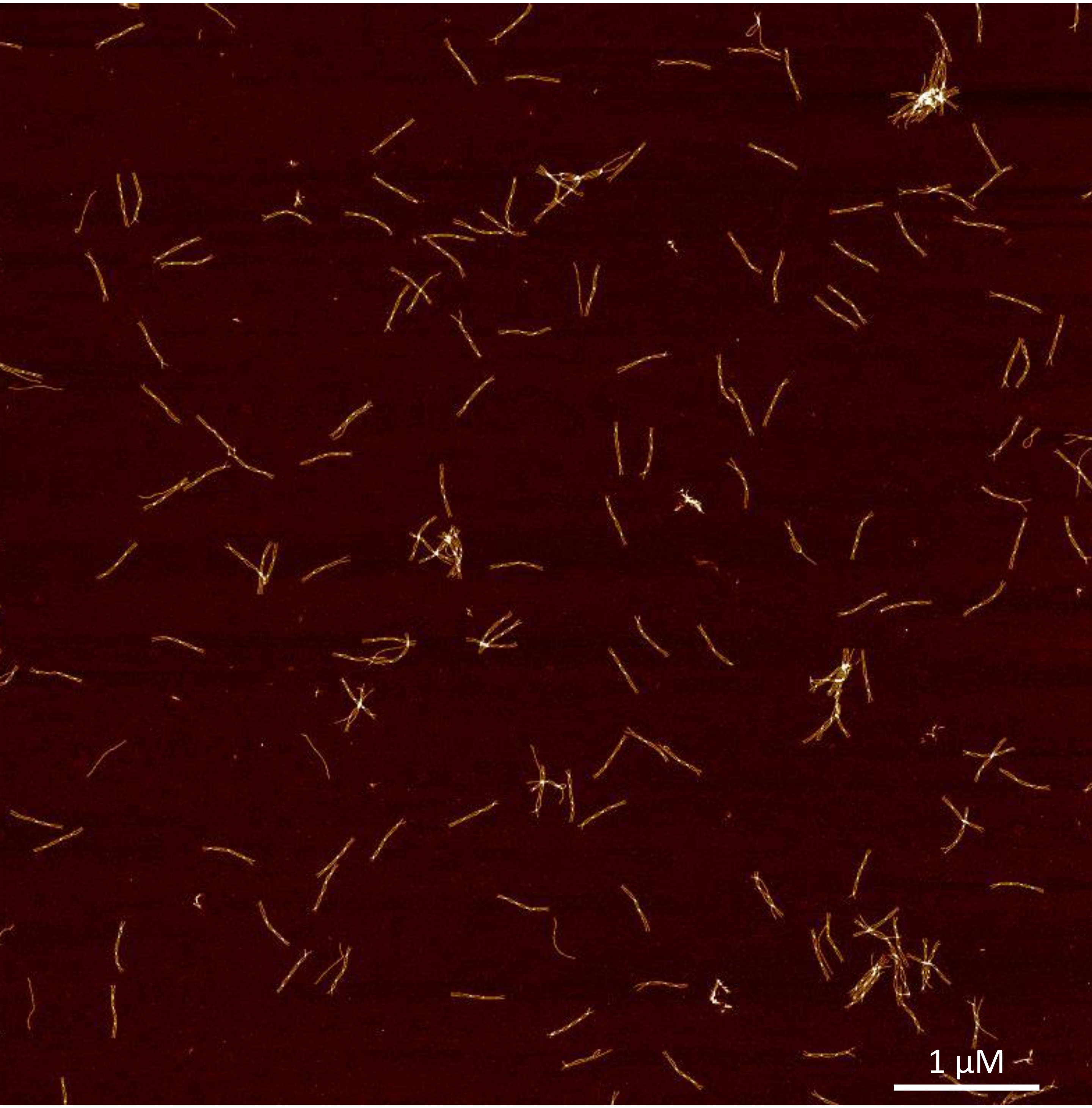

Supplement: Supplementary file 1 — Supporting Information [file 41598_2017_7796_MOESM1_ESM.pdf]
